# Pirfenidone regulates LPS mediated activation of neutrophils

**DOI:** 10.1038/s41598-020-76271-3

**Published:** 2020-11-17

**Authors:** Shankar J. Evani, S. L. Rajasekhar Karna, Janakiram Seshu, Kai P. Leung

**Affiliations:** 1 Division of Combat Wound Repair, U.S. Army Institute of Surgical Research, 3698 Chambers Pass, Building 3610, JBSA Fort Sam Houston, San Antonio, TX 78234-7767 USA; 2 South Texas Center for Emerging Infectious Diseases (STCEID) and Department of Biology, The University of Texas at San Antonio, One UTSA Circle, San Antonio, TX USA

**Keywords:** Mechanisms of disease, Cell biology

## Abstract

Excessive inflammation or its absence may result in impaired wound healing. Neutrophils are among the first innate immune cells to arrive at the injury site. They participate in infection control and debris removal to initiate healing. If not timely resolved, neutrophils can cause excessive tissue inflammation and damage. Drugs with anti-inflammatory and anti-fibrotic effects are of promise for improving healing by balancing the primary defensive functions and excessive tissue damage actions. Of interest, pirfenidone (Pf), an FDA approved anti-fibrotic drug to treat idiopathic pulmonary fibrosis, has been shown to ameliorate inflammation in several animal models including mouse deep partial-thickness burn wounds. However, there is a lack of mechanistic insights into Pf drug action on inflammatory cells such as neutrophils. Here, we examined the treatment effects of Pf on LPS-stimulated neutrophils as a model of non-sterile inflammation. Firstly, Pf reduced chemotaxis and production of pro-inflammatory ROS, cytokines, and chemokines by LPS-activated neutrophils. Secondly, Pf increased anti-inflammatory IL-1RA and reduced neutrophil degranulation, phagocytosis, and NETosis. Thirdly, Pf affected downstream signaling kinases which might directly or indirectly influence neutrophil responses to LPS. In conclusion, the results suggest that Pf lessens the inflammatory phenotypes of LPS-activated neutrophils.

## Introduction

Neutrophils, the most abundant white blood cells, are one of the body’s main cellular components of innate immunity. In response to infection or tissue injury, neutrophils rapidly infiltrate into damaged tissue to remove debris and microbes. At the site, neutrophils release multitude of cytokines (e.g., Interleukin-1β/IL-1β and Tumor Necrosis Factor-α/TNFα) and chemokines (e.g., IL-8 and monocyte chemoattractant protein-1/MCP1) to propagate the inflammatory response. The cells undergo respiratory burst to produce reactive oxygen species (ROS; singlet oxygen, superoxide, and hydroxyl radical) that kill microbes. Via degranulation, they release antimicrobial peptides (e.g., LL37 and defensins) and degradative enzymes (e.g., cathepsin G, matrix metalloproteinase (MMP), and elastase) to clear infection and degrade various tissue components to facilitate healing^1^. Neutrophils phagocytose the invading microbes and also release de-condensed chromatin and granular contents to extracellular space called neutrophil extracellular traps (NETS) to further incapacitate them^2^.

These processes, which rapidly curtail infections or contain tissue injuries, are thought to be directed by evolution to rapidly eliminate microbes at the expense of indiscriminate damage to host cells and tissue^1,3^. In the absence of efficient and timely resolution, unwarranted consequences such as uncontrolled inflammation and extensive tissue damage result in poor tissue healing^3,4^.

Tissue fragments and cellular debris (Damage Associated Molecular Patterns: DAMPs) from injuries/infections (Pathogen Associated Molecular Patterns: PAMPs) exacerbate inflammation by hyper-activating immune cells through pattern recognition receptors, triggering activation of Nuclear factor kappa-light-chain-enhancer of activated B cells (NF-κB) and other transcription factor^5^. Hyper-activation of neutrophils and the resulting uncontrolled inflammation have been shown to hinder tissue recovery post infection or injury by preventing re-epithelialization and promoting scar-tissue formation^6–8^. In burn wounds, exacerbated or prolonged pro-inflammatory responses in tissue are associated with increased fibrosis resulting in keloids and hypertrophic scars^9^. These observations underscore the need for balancing tissue inflammation and reparative responses in order to minimize excessive tissue damage for improved healing outcomes.

Suppressed inflammation has been associated with reduced tissue damage and improved wound healing. Martin et al. showed that PU.1 null mouse lacking macrophages and neutrophils, which is incapable of mounting an inflammatory response, repairs skin wounds with reduced scarring similar to that of scar-less fetal wound healing^10^. Similarly, through the use of anti-neutrophil antibodies, Dovi et al. demonstrated that neutrophil-depleted mice heal faster than control mice^11^. They further observed accelerated healing in diabetic mice devoid of neutrophils^11^. Suppression of neutrophil-dependent tissue damage can have the side effect of diminished protection from infection; but with the multitude of antimicrobial treatments available today, infection management is possible. Therefore, this provides potential opportunities for selective therapeutics that target neutrophils to restrain inflammation and its tissue-damaging effects for improved healing outcomes^1^.

One therapeutic drug with anti-inflammatory and antifibrotic activities that we have been studying as a potential wound healing treatment option is pirfenidone (Pf). Pf is a modified pyridine small molecule (185.22 g/mol) approved for use in treating idiopathic pulmonary fibrosis^12,13^. Recently, we showed that Pf lessens the profibrotic phenotype of transforming growth factor-β1 (TGF-β1)-stimulated human dermal myofibroblasts by decreasing collagen deposition, reducing fibrosis-related gene expression^14^, and lowering contractility in vitro^15^. We also tested the ability of Pf to improve burn wounds and scarring. We showed that Pf treatment of mouse deep partial-thickness burn wounds during the inflammatory phase of healing reduces tissue inflammation with decreased presence of pro-inflammatory cytokines, chemokines, neutrophil infiltration, and trends to reduce collagen deposition in wounds^16^.

Furthermore, multiple other studies and clinical trials have also shown that Pf elicits its anti-inflammatory and anti-fibrotic potential by down-regulating ROS generation, TGF-β expression, chemokine C–C motif ligand-18 (CCL18) production, MMP generation, and pro-fibrotic cytokine and chemokine production^17^. These molecular events further attenuate pro-fibrotic events such as fibroblast-myofibroblast transformation^18^, collagen production, inflammation, and pro-fibrotic macrophage recruitment in pulmonary and other models of fibrosis of skin, kidneys, and other tissues^19^. Pf was also shown to lower immune cell accumulation, macrophage differentiation to M2 phenotype, and pro-inflammatory and angiogenic cytokine production^20,21^.

However, among these studies, several questions remain unanswered. For example, it is unclear about the effects of Pf on the inflammatory phenotypes and functions of neutrophils during the treatment. To address this question, we chose to use lipopolysaccharide (LPS)-activated neutrophils as the inflammatory surrogate for understanding the mechanistic insights into Pf drug action on modulation of neutrophil activation.

Most tissue injuries are inevitably contaminated with microbes. It is well established that PAMPs play a critical role in mounting inflammatory responses through their interactions with surface-expressed toll-like receptors (TLRs) in innate immune cells such as neutrophils and macrophages^22^. LPS, a well-characterized PAMP, is abundantly present in the outer membrane of Gram-negative bacteria and has been extensively used as an initiator of inflammatory environment in multiple in vitro and in vivo inflammation models^23^. LPS is recognized by the membrane bound TLR4-myeloid differentiation factor 2 (MD2) complex and their interactions lead to activation of multiple signaling molecules and subsequent production of pro-inflammatory mediators^24^.

Using LPS-activated human neutrophils, we sought to test the impact of Pf on production of pro-inflammatory mediators, oxidative burst, degranulation, phagocytosis, and NETosis that are carried out by these cells upon LPS stimulation. We found that Pf treatment of LPS-activated neutrophils at 0.5 mg/ml decreased chemotactic responses to *N*-formylmethionyl-leucyl-phenylalanine (fMLP), leukotriene B4 (LTB4), and IL-8, and their release of pro-inflammatory ROS, cytokines (TNFα, IL-1β, and IL-6), chemokines (IL-8, MCP-1, and macrophage inflammatory proteins-1α/MIP-1α). Also, similar Pf treatment increased anti-inflammatory IL-1 receptor antagonist (IL-1RA) release. In contrast, Pf treatment at 0.5 mg/ml transiently dampened neutrophil antimicrobial functions such as degranulation, phagocytosis, and NETosis. Cumulatively, Pf can potentially suppress tissue inflammation that is harmful for proper healing of injured tissues with a transient decrease in antimicrobial functions.

## Results

### Pf did not affect the neutrophils cell viability at early time points of drug treatment

**Figure 1:**
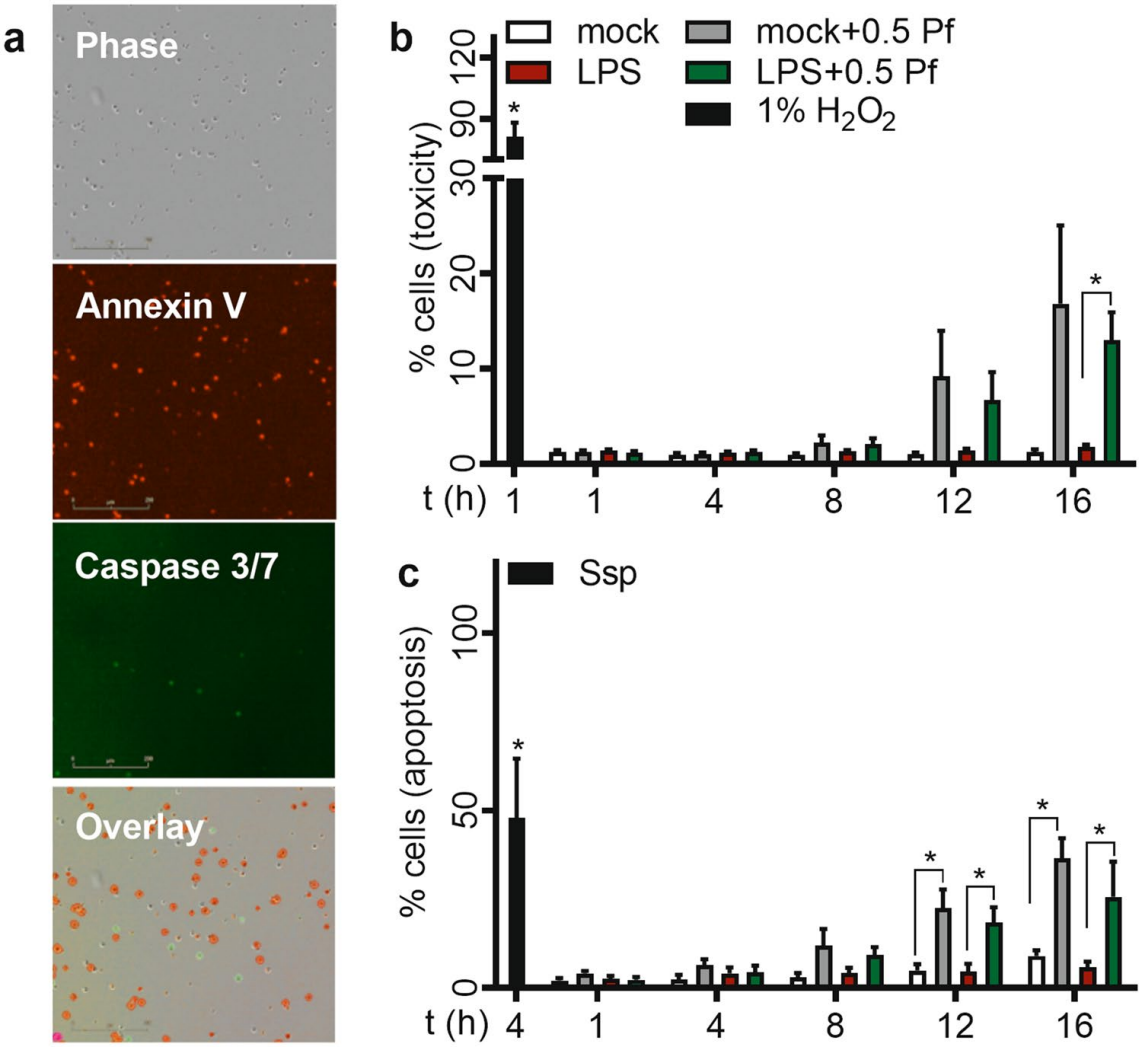
Effect of Pf on neutrophil cell health. Media alone (mock) or 20 ng/ml of LPS (LPS)-activated neutrophils were treated with 0 or 0.5 mg/ml Pf (+ Pf) along with (**b**) Cytotox-red or (**a**, **c**) Annexin V-red and Caspase-3/7-green to measure toxicity or apoptosis respectively by live cell fluorescent imaging of events using Incucyte S3 system. H_2_O_2_- and Ssp (staurosporine)-treated neutrophils were used as positive controls for measuring necrosis/toxicity and apoptosis respectively. Representative individual images with neutrophils double stained with AnnexinV—red and caspase3/7—green along with overlay-image is shown (**a**). Data points in the graphs show the percentage of positive dead cells (**b**) and apoptotic cell percentage (**b**) calculated from mean ± SEM of 6 different experiments done in triplicates. *Represents significant difference (p-value ≤ 0.05; two-way ANOVA) between various treatment groups.

To understand drug toxicity profile, we evaluated viability and apoptosis in Pf treated-neutrophils by utilizing the Incucyte live-cell imaging platform. Similar to Pf’s toxicity effects on other cell types, drug induced toxicity is time (Fig. 1b) and concentration (Supplementary Figure S2) dependent. Here, LPS didn’t significantly alter neutrophil viability though the mean value of this group at 16 h appears to trend lower than mock treated neutrophils. Pf at 0.5 mg/ml did not significantly alter cell death in mock neutrophils but increased the cell death of LPS-treated neutrophils at 16 h post treatment. The mean values of percentage cell death in both mock and 0.5 mg/ml Pf treated neutrophils were at similar levels till about 8 h post treatment (Fig. 1b). Similarly, neutrophil apoptosis (measured by annexin V and caspase 3/7 positive staining) significantly increased in Pf treated conditions with respect to untreated controls in both mock and LPS activated conditions starting from 12 h post treatment (Fig. 1c). Pf at 0.5 mg/ml did not induce apoptosis up to 4 h post treatment (Fig. 1c). For the positive controls, One % hydrogen peroxide and staurosporine (ssp) treatment induced very high levels of cell toxicity and apoptosis respectively. Recent evidences suggest a longer neutrophils life cycle^25^. Also, Matrigel adherent neutrophils may possibly show low apoptosis/cell death than normally observed. Overall, Pf at 0.5 mg/ml did not induce toxicity in neutrophils up to 12 h post treatment. On contrary, similar Pf treatment caused a gradual increase in apoptosis with time starting at 8 h post treatment.

### Pf treatment decreased neutrophil chemotaxis

**Figure 2:**
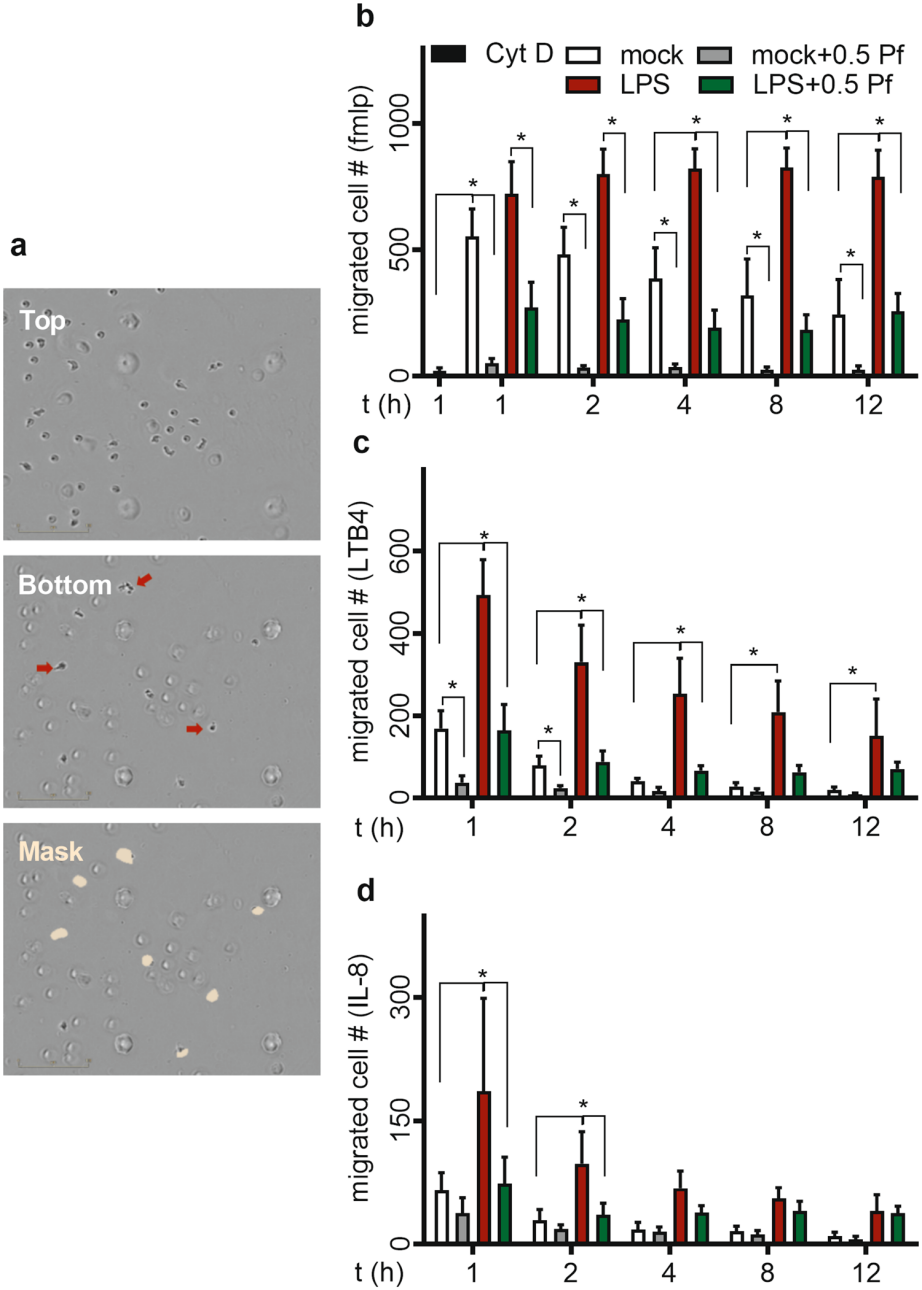
Pf reduces chemotactic migration of neutrophils. Mock or LPS-activated neutrophils were treated with ± 0.5 mg/ml Pf and their migration towards chemo-attractants fMLP (300 nM), LTB4 (100 nM) and IL-8 (100 ng/ml) were measured in a trans-well assay system by Incucyte S3 live cell imaging system. Representative images with neutrophils in top well and migrated cells in bottom trans-well and efficiency of mask in identifying migrated cells (red arrow pointing migrated cells) is shown (**a**). Cytochalasin-D (cytD)-treated neutrophils were used as a negative control for neutrophil chemotaxis. The graphs represent the time course of migrated neutrophil number in response to chemotactic agents fMLP (**b**), LTB4 (**c**), IL-8 (**d**). Data points were calculated from mean ± SEM obtained from 6 different experiments done in triplicate. *Represents significant difference (*p*-value ≤ 0.05; two-way ANOVA) between various treatment groups.

Here we tested the chemotactic migration of Pf (±) and LPS (±) treated cells to potent chemo-attractants which included fMLP, LTB4, IL-8 and TGF-β. These chemo-attractants showed a rank order of potency—fMLP > LTB4 > IL-8 > TGFβ—for inducing chemotactic migration of mock or LPS-stimulated neutrophils (Fig. 2). In negative control group, cytochalasin-D (cyt-D) treatment substantially decreased neutrophil chemotaxis to fMLP (Fig. 2b). TGF-β1 showed minimum to no chemotaxis (data not shown). LPS significantly increased neutrophil chemotaxis respective to mock controls to all chemo-attractants. A majority of neutrophils migrate towards chemoattractant in the first 1 to 2 h under almost all test conditions with the exception that the LPS-treated neutrophils displayed a different time response when fMLP was used as the chemoattractant. Pf (0.5 mg/ml) treatment of LPS-activated neutrophils significantly decreased the chemotaxis induced by fMLP (Fig. 2b), LTB4 (Fig. 2c), and IL-8 (Fig. 2d) of both LPS and mock treated neutrophils. In the presence of Pf, mock treated neutrophils too showed very low migration (Fig. 2).

### Pf decreased neutrophil free radical production

**Figure 3:**
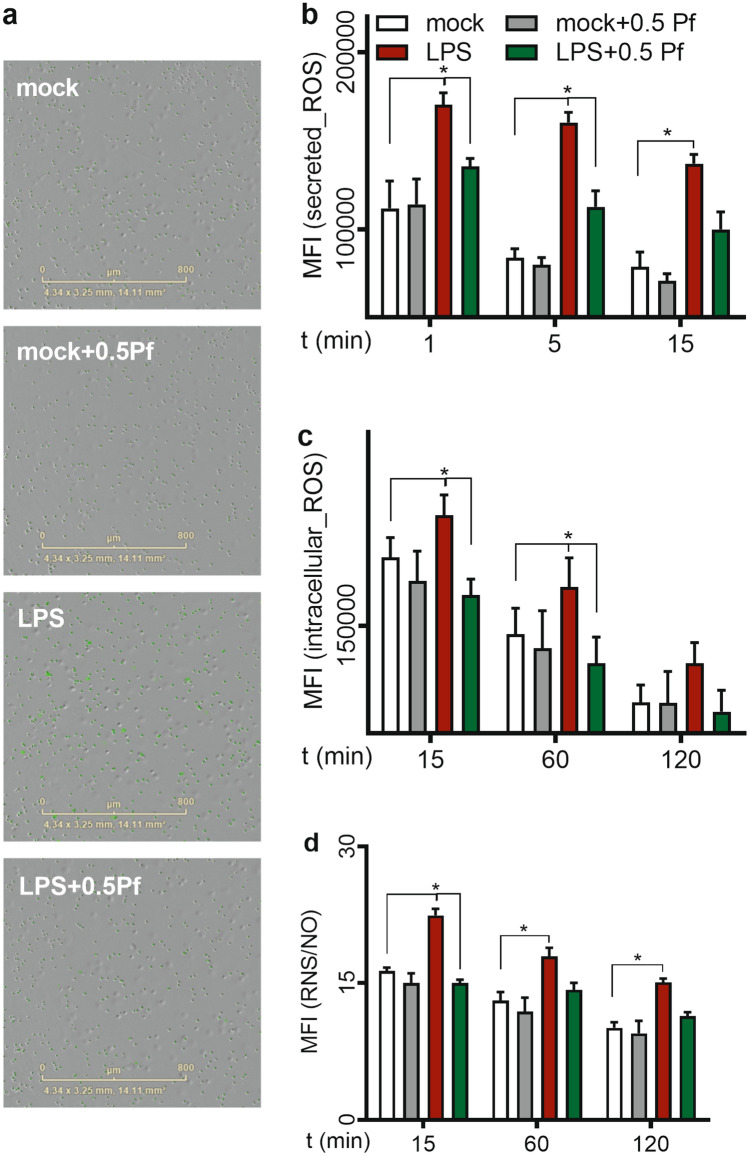
Pf reduces neutrophil oxidative burst. OxyBURST Green H2HFF BSA (extracellular/secreted ROS; b), CellROX Green (intracellular ROS; **a**,**c**) and DAF-FM Diacetate (total RNS /NO; **d**) dyes were used to measure free radical production from neutrophils (mock or LPS-activated conditions neutrophils treated with ± 0.5 mg/ml Pf). Representative images from Incucyte system showing intracellular ROS levels (green fluorescence) for different conditions at 1-h time point is shown here (**a**). The graphs with mean fluorescent intensity (MFI) data represent the time course of respective free-radical production by neutrophils (normalized). Data points were calculated from mean ± SEM obtained from 6 different experiments done in triplicates. *Represents significant difference (p-value ≤ 0.05; two-way ANOVA) between various treatment groups.

We determined the effect of Pf on neutrophil reactive oxygen and nitrogen species production. Compared to the mock control, LPS triggered ROS and reactive nitrogen species (RNS)/NO production (Fig. 3) as seen in both of released ROS (Fig. 3b), intracellular accumulation of ROS (Fig. 3c), and total RNS/NO (Fig. 3d). Naïve neutrophils attached to Matrigel showed release of ROS and deposition of ROS and RNS/NO (Fig. 3b–d respectively). Because oxidative burst is a fast responding event to stimuli in neutrophils; therefore, we observed higher levels of ROS and RNS/NO release in each test condition at early time points. These responses are trending downwards as time proceeds. Pf at 0.5 mg/ml decreased the cellular deposition (Fig. 3c) and release (Fig. 3b) of ROS significantly by at least 20%. RNS/NO production was also lowered due to the drug action (Fig. 3d). Also, we observed similar changes when these cells were tested for fluorescence intensity by flow cytometry (Supplementary Figure S3).

### Pf dampened neutrophil pro-inflammatory response

**Figure 4:**
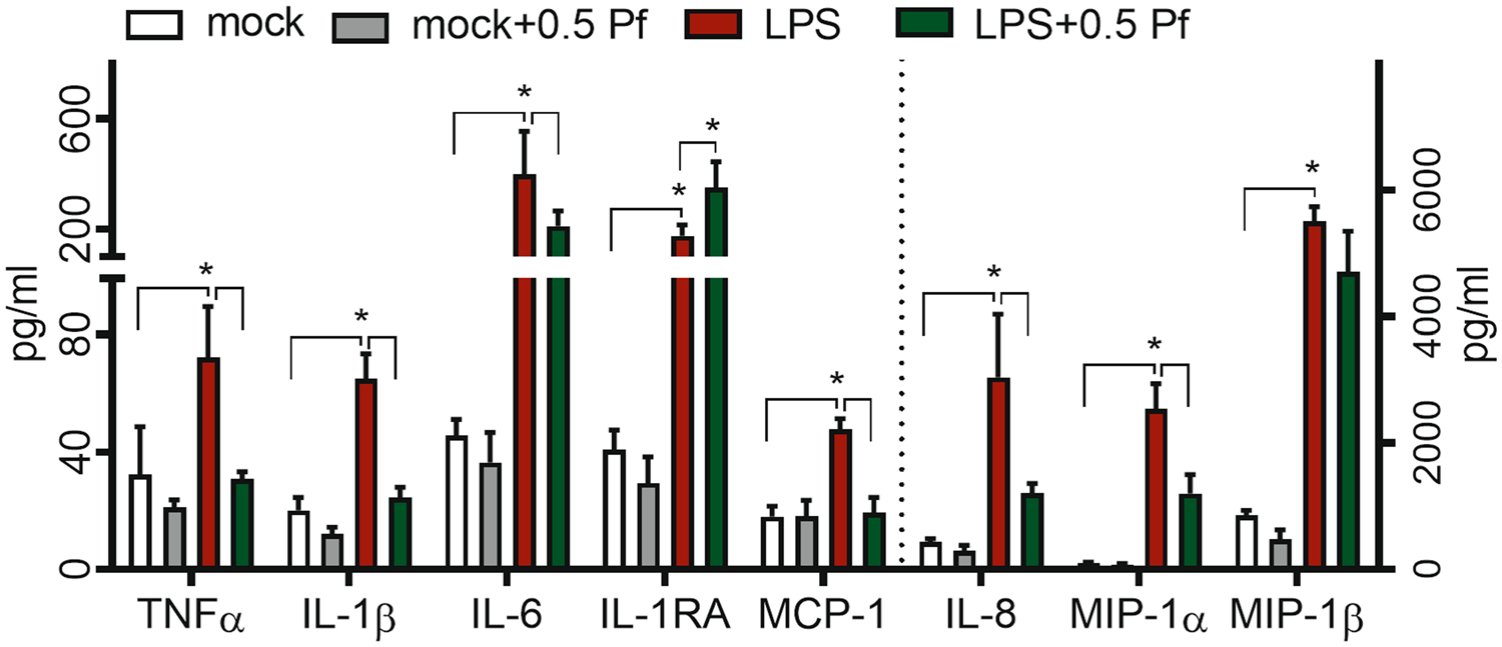
Pf modulates neutrophil pro-inflammatory response. Mock or LPS-activated neutrophils were treated with ± 0.5 mg/ml Pf and incubated for 4 h. Supernatants were collected and quantified for inflammatory mediators secreted by neutrophils using Procarta multiplex assays. Data points in the graphs representing mean ± SEM values obtained from 6 different experiments done in triplicate. *Represents significant difference (*p*-value ≤ 0.05; two-way ANOVA) between various treatment groups.

Compared to mock control, LPS significantly induced the release of pro-inflammatory cytokines TNFα, IL-1β, and IL-6 as well as chemokines MCP-1, IL-8, MIP-1α, and MIP-1β from neutrophils. Pf treatment (0.5 mg/ml) significantly reduced pro-inflammatory cytokines TNFα (~ 57%), IL-1β (~ 62%) and IL-6 (~ 47%) and chemokines MCP-1 (~ 60%), IL-8 (~ 60%), and MIP-1α (~ 52%) in LPS-treated neutrophils whereas mock controls showed no change in these responses upon treatment with Pf. Pf treatment of LPS-stimulated neutrophils also increased the release of IL-1RA (~ 100%), an anti-inflammatory IL-1 receptor antagonist (Fig. 4). Interestingly, in addition to the ability to reduce the release of pro-inflammatory cytokines and chemokines, Pf was able to stimulate the expression of anti-inflammatory IL-1RA in LPS-activated neutrophils. We observed higher inflammatory TNFα and IL-8 production in neutrophils (high purity > 99.3%) treated with tenfold higher concentration of LPS (200 ng/ml) and Pf also successfully reduced the levels of these mediators suggesting a potent anti-inflammatory effect of Pf (Supplementary Figure S4). The anti-inflammatory activity at other Pf concentrations used in the study (0.1 and 1 mg/ml Pf) for up to 16 h post treatment (Supplementary Table S1 and S2). Further, Pf successfully suppressed inflammatory TNFα, and IL8 in highly activated neutrophils activated with higher concentration (200 ng/ml) of LPS (Supplementary Figure S4). Similar drug action was seen when using neutrophils with higher purity (> 99%; Supplementary Figure S4).

### Pf decreased neutrophil degranulation

**Figure 5:**
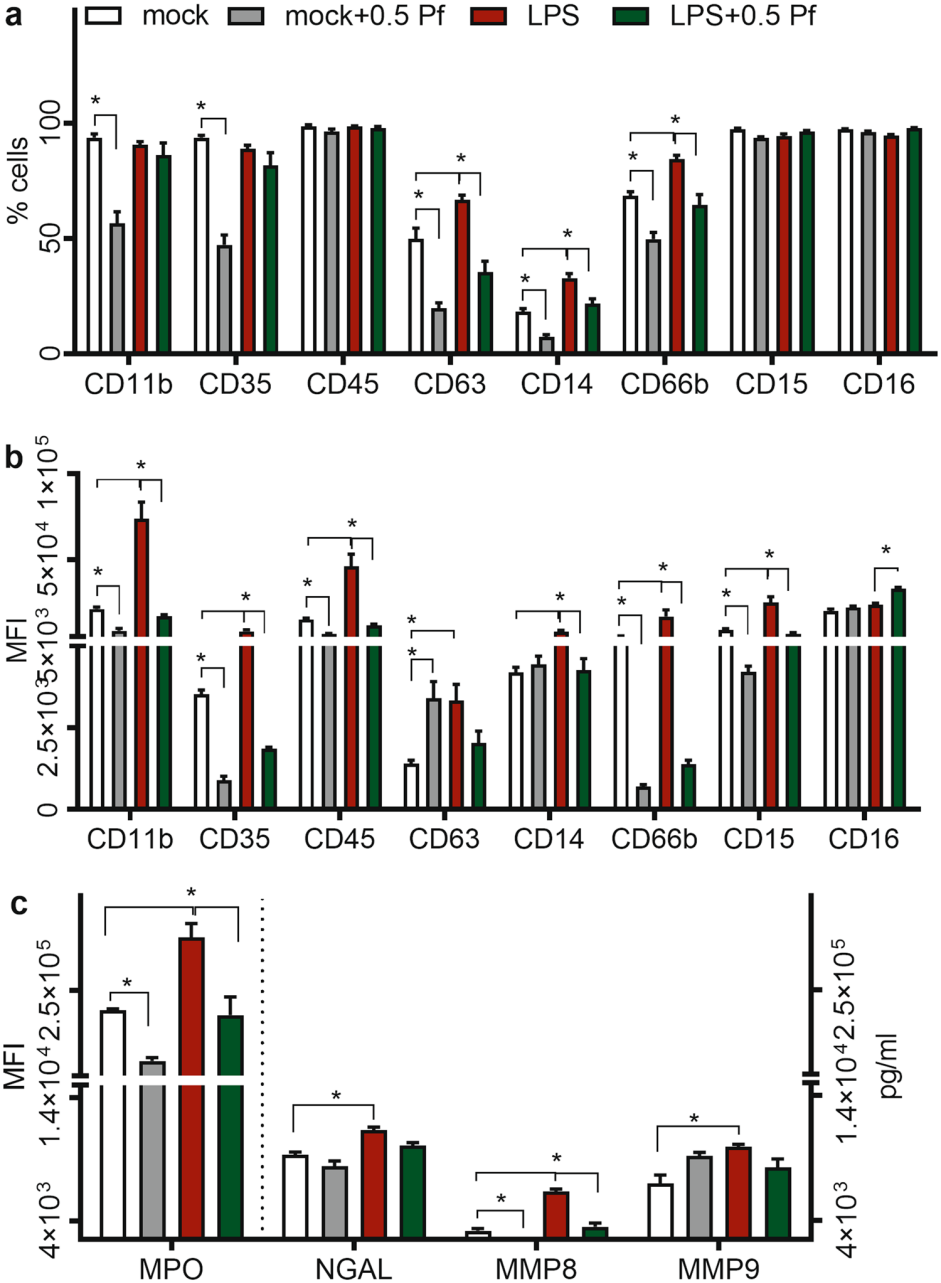
Pf decreases neutrophil degranulation. Mock or LPS-activated neutrophils were treated with ± 0.5 mg/ml Pf and incubated for 4 h. Cells and supernatants were collected and analyzed for degranulation markers and proteins. Cells were stained for degranulation markers using fluorescent antibodies and quantified by flow cytometry for % cells (**a**) and MFI (**b**). Supernatants were analyzed for MPO and quantity of degranulation proteins (MMP8, MMP9, and NGAL) by fluorescent MPO assay and Procarta-Multiplex Immunoassay respectively (**c**). Data points in the graphs represent mean ± SEM values obtained from 6 different experiments done in triplicate. *Represents significant difference (*p*-value ≤ 0.05; two-way ANOVA) between various treatment groups.

To understand the effect of Pf on degranulation, at 4 h post treatment, we tested changes in multiple markers, both cellular and released products that are representing various types of neutrophil granules. LPS stimulated degranulation which was represented by the increase in percentage of cells expressing Cluster of differentiation 14 (CD14), CD63, CD66b (Fig. 5a). Further, compared to mock controls, upon treatment with LPS there was a significant increase in mean intensity for surface expression markers CD11b, CD14, CD35, CD45, CD63, CD66b and CD15 (Fig. 5b). Pf treatment decreased the percentage of cells expressing CD11b, CD14, CD35, CD63, CD66b & CD15 in mock treated neutrophils, and CD14, CD63 & CD66b in LPS-treated neutrophils (Fig. 5a). Additionally, Pf decreased the mean intensities of CD11b, CD14, CD35, CD45, CD66b, and CD15 in both mock (with the exception of CD14) and LPS-treated neutrophils (Fig. 5b). Histograms for the respective degranulation markers on cell surface are shown in Supplementary Figure S5 for comprehensive understanding of the treatment effect. Compared to mock group’s direct measurement of degranulation products such as MMP8, MMP9, neutrophil gelatinase-associated lipocalin (NGAL), and myeloperoxidase (MPO) revealed an increase in neutrophil degranulation upon LPS treatment. Pf treatment lessened the increase of MMP8 and MPO levels in both mock and LPS-treated neutrophils (Fig. 5c). Also, we observed anti-degranulation potential of Pf even at higher activation state of neutrophils (Supplementary Figure S4). The transient decrease in neutrophil antimicrobial functions were seen at other Pf concentrations used in the study (0.1 and 1 mg/ml Pf) for up to 16 h post treatment (Supplementary Table S1 and S2). Also, Pf reduced CD11b expression and MPO secretion (representative degranulation markers) in 200 ng/ml of LPS activated neutrophils (Supplementary Figure S4). We observed similar drug action when using neutrophils with higher purity (> 99%; Supplementary Figure S4).

### Pf decreased neutrophil phagocytosis

**Figure 6:**
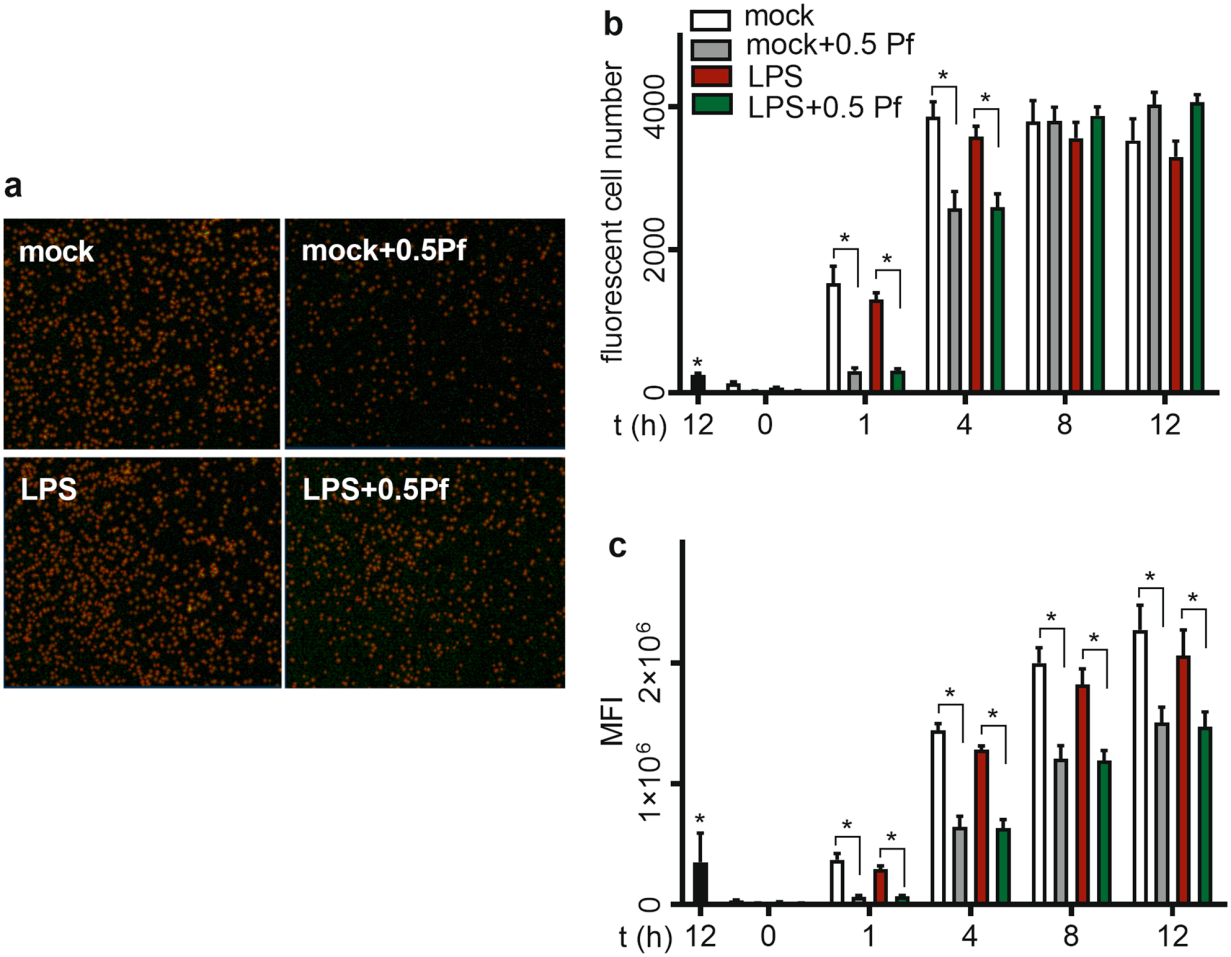
Pf decreases phagocytosis of E. coli by neutrophils. Mock or LPS-activated neutrophils were treated with ± 0.5 mg/ml Pf along with the recommended concentration of pHrodo E. coli Bio Particles. Plates were placed in the Incucyte S3 for live cell fluorescence imaging for 12 h and analyzed for phagocytosis. (**a**) Representative images of different conditions at 1-h time point is shown here. Images were analyzed by the Incucyte analysis software for fluorescent cell number (**b**) and MFI (**c**) of phagocytosis. CytD (solid black bar) treated neutrophils were used as a negative control of phagocytosis. Data points in the graphs represent mean ± SEM values obtained from 6 different experiments done in triplicates. *Represents significant difference (p-value ≤ 0.05; two-way ANOVA) between various treatment groups.

To test the phagocytic potential of neutrophils in various treatment profiles, we used pH sensitive *E. coli* bio-particles and measured phagocytosis by fluorescence quantification upon acidification. Regardless of treatment groups, we observed that phagocytosis increased over time in respect to percentage of cells participating in phagocytosis (Fig. 6b) and the mean fluorescence intensity (indicative of intracellular *E. coli* load) per cell (Fig. 6c). We detected no significant difference in phagocytosis profiles between mock and LPS treated neutrophils. However, upon Pf treatment there was a significant drop in the percentage of cells phagocytosing the fluorescent-labeled *E. coli* initially but slowly recovered with time to the similar level of cells with no Pf treatment (Fig. 6b). Likewise, we observed that the intensity of phagocytosis was significantly lower in cells with Pf treatment to that of no treatment (Fig. 6c). There was also a time-dependent recovery of fluorescent intensity in Pf-treated cells (mock and LPS-activated).

### Pf decreased neutrophil NETosis

**Figure 7:**
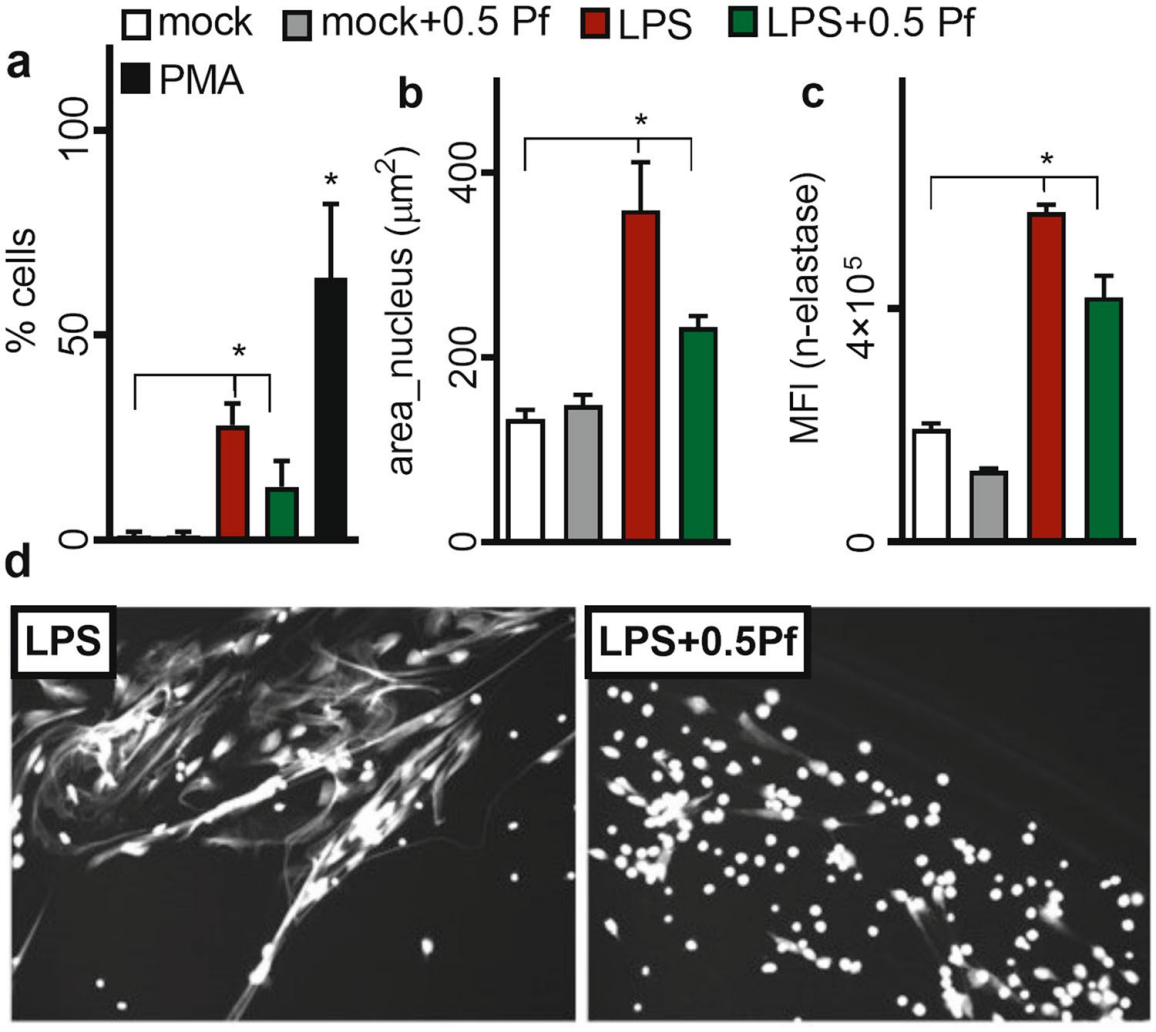
Pf reduces NETosis of neutrophils. Media alone or LPS-O128- (40 μg/ml) activated neutrophils were treated with ± 0.5 mg/ml Pf for 4 h (**a**–**c**). Cells were fixed and stained for N-Elastase (in NETs) and nucleus (DAPI) without cell permeabilization. Fluorescent images were taken by a Cytation 5 cell imaging reader and analyzed for NETosis. Percent NETosis (**a**), average nucleus area (**b**), N-Elastase MFI (**c**) are shown. PMA treated neutrophils were used as a positive control for neutrophil NETosis. Data points in the graphs represent mean ± SEM values obtained from 6 different experiments done in triplicates. *Represents significant difference (p-value ≤ 0.05; two-way ANOVA) between various treatment groups. Representative grey scale images with DAPI stained neutrophil NETs in LPS ± Pf treated neutrophils are shown (**d**).

As shown in Fig. 7, LPS induced neutrophil NETosis, while mock controls or the Pf treated alone did not show NETosis. LPS stimulation showed increased percent of -activated neutrophils displaying NETosis phenotype (Fig. 7a) with the increase in nucleus size (Fig. 7b) and NET related-elastase (Fig. 7c). PMA control showed high levels of NETosis (Fig. 7a). Pf treatment (0.5 mg/ml) of LPS-activated cells showed a significant reduction of % NETosis, nuclear size, and elastase intensity (Fig. 7).

### Pf down-regulated multiple kinases to modulate neutrophil response to LPS

**Figure 8:**
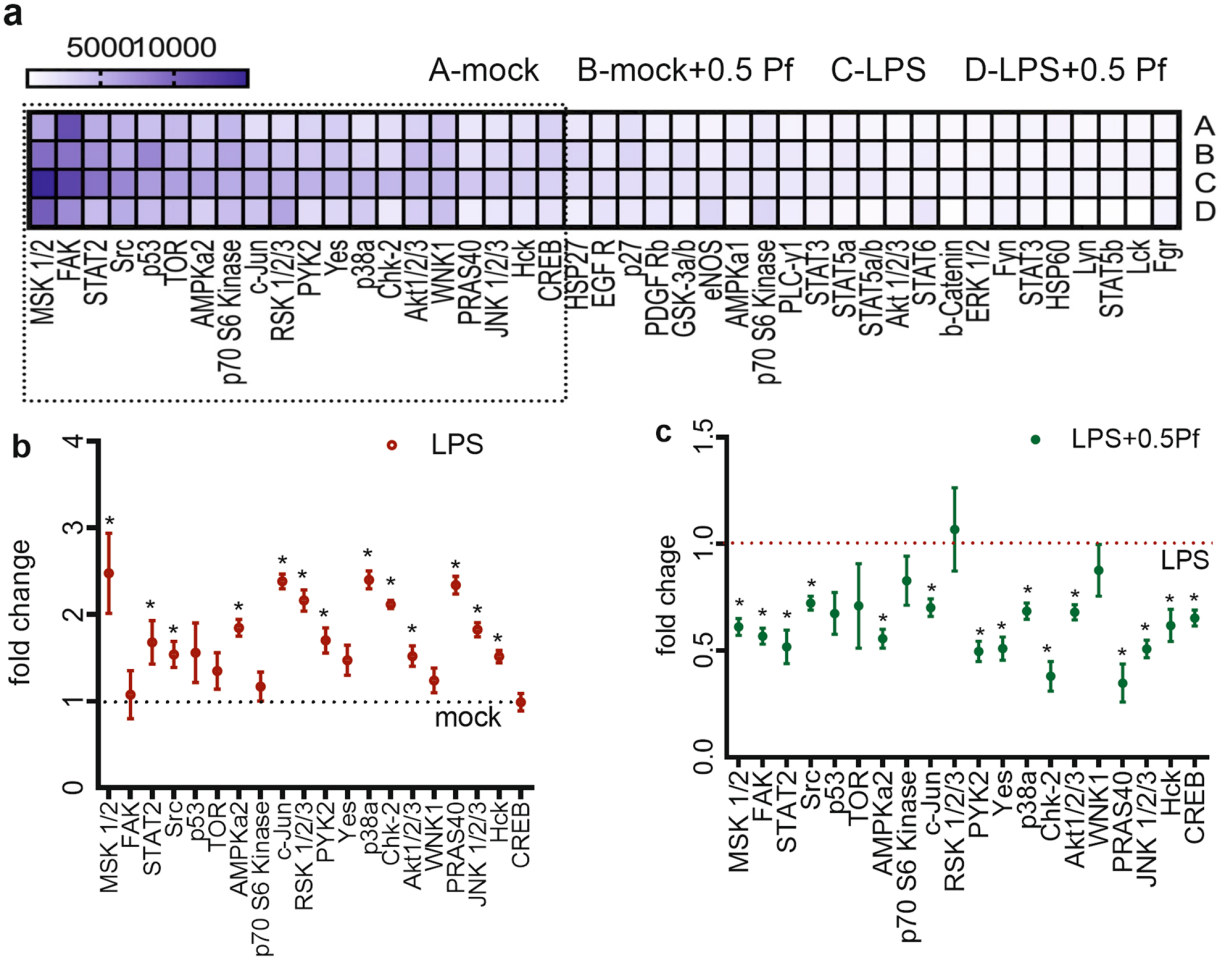
Pf down-regulates kinases to modulate multiple-functional changes in neutrophils. Media alone or LPS-activated neutrophils were treated with ± 0.5 mg/ml Pf (**a**–**c**) for 3 h (**a**–**c**). Cells were lysed and assayed for phospho-kinase signaling proteins by a Proteome Profiler Array Kit. The heat map of normalized mean chemiluminescence units as a measure of protein density/quantity from 6 donors (**a**). The dotted line depicts the cutoff, above which represents phosphorylated proteins expressing ≥ 3000 chemiluminescence units and are selected for further analysis in (**b**) and (**c**). (**b**) Data points in the graph represent the fold change of protein expressed in LPS-treated neutrophils with respect to the mock treated neutrophils (mock = 1). (**c**) Data points in the graph represents the fold change of proteins expressed in LPS-activated neutrophils with Pf treatment (0.5 mg/ml) relative to untreated LPS-activated neutrophils without Pf treatment (LPS = 1). Data in the graphs was plotted from mean ± SEM values calculated from 6 different experiments. *Represents significant difference (p-value ≤ 0.05; ANOVA) between treatment groups.

LPS triggered a slew of functional responses which included oxidative burst, pro-inflammatory response and degranulation along with heightened chemotactic migration. To assess the regulation of LPS mediated responses by Pf at the molecular level, we performed the phospho-kinase pathway array. We observed that at 3 h post treatment LPS upregulated phosphorylation of multiple downstream kinases of NF-κB pathway. Compared to mock controls, mitogen- and stress-activated kinase (MSK1/2), p38αMAPK, protein kinase B (Akt), ribosomal s6 kinase (RSK), PYK2, c-Jun N-terminal kinase (JNK), proline-rich Akt substrate of 40 kDa (PRAS40), AMP-activated protein kinase a (AMPKa), checkpoint kinase 2 (Chk-2), signal transducer and activator of transcription 2 (STAT2), Src and c-Jun showed significantly higher phosphorylation in LPS-treated neutrophils (Fig. 8a,b). Theses kinases showed a minimum expression of 3000 chemiluminescence units signifying their activation (Fig. 8a). Pf treatment decreased the phosphorylation levels of pro-inflammatory mediators and degranulation regulators such as p38α, MSK1/2, Akt, hematopoietic cell kinase (Hck), JNK, c-jun, PYK2, target of rapamycin (TOR), PYK2 and cAMP Response Element-Binding Protein (CREB).

## Discussion

Pf is an approved therapy for the treatment of idiopathic pulmonary fibrosis^26^. Owing to its broad action such as anti-oxidative, anti-fibrotic and anti-inflammatory activities, Pf is also a potential therapeutic for the treatment of fibrosis/scarring in graft rejection, severe skin burns, heart, liver, and kidney ailments^27^. We have been studying the repurposing of Pf for prevention and treatment of burn-induced hypertrophic scarring, a hallmark of burn injury likely due partly to excessive and prolonged inflammation^16,18^. To better understand the anti-inflammatory property of Pf, we employed an in vitro model wherein LPS-stimulated human neutrophils were used for testing the anti-inflammatory potential of Pf. We chose LPS-activated neutrophils because the system closely mimicked the activation of innate immune cells and their pro-inflammatory responses during the initial phases of infection or injury.

Pf has a well-established safety and tolerability profile^26^. Even at a very high treatment dose of 2403 mg per day for patients with pulmonary fibrosis for an extended period, Pf showed minimal treatment adverse effects^28^. Previous in vitro studies on various cell types including fibroblasts, macrophages and epithelial cells demonstrated that these cells can tolerate different Pf concentrations ranging from 0.2 to 2 mg/ml^20,29,30^. Similarly, neutrophils showed a moderate tolerance to Pf up to 0.5 mg/ml with low levels of apoptosis and cell death. However, irrespective of the activation state of neutrophils, Pf at 1 mg/ml caused significant neutrophil apoptosis/cell death in vitro (Supplementary Figure S2) and may interfere with any functional observations made in vitro. Hence, this concentration of Pf was not used in this study in the context of human neutrophils. Our results did confirm that, upon Pf treatment at 0.5 mg/ml, neutrophils did not undergo immediate necrotic cell death, allowing us to characterize Pf treatment effects on the inflammatory properties of LPS-activated neutrophils.

Necrotic cell death due to injury and infection results in the concomitant release of DAMPs- and PAMPs-induced byproducts such as LTB4 and formyl peptides. These chemoattractants, in combination with subsequent chemokine C–X–C motif ligand 8 (CXCL8/IL-8) released from resident macrophages and fibroblasts, trigger neutrophil influx to the wound site^31,32^. Excessive infiltration of neutrophils together with their prolonged presence and activation are seen in burn and chronic non-healing wounds^22,33^. This excessive post-traumatic inflammation may result in a positive feedback loop of pro-inflammatory events which further aggravate tissue damage in the wound bed^8,34^. In our studies, we observed increased chemotactic migration of LPS-activated neutrophils at different degrees towards fMLP, IL8, and LTB4. Upon Pf treatment of these neutrophils, we saw a drastic reduction in neutrophil migration towards these potent chemoattractants. The results suggest that Pf can influence the chemotactic migration of neutrophils to the wound site and hence may reduce inflammation. Neutrophils which reach the tissue may trigger excessive inflammation as a bystander effect due to exacerbated free radical, cytokine, and chemokine production.

Tissue influx of neutrophils and their encounter with DAMPs, microbes, or microbial PAMPS trigger oxidative burst with immediate release of free radicals to curtail infection. Heightened free-radicals produced in the tissue by resident cells and infiltrating immune cells cause oxidative stress resulting in severe tissue damage. These ROS and RNS free radicals play a major role in inducing fibrosis by exacerbating inflammation, neutrophil chemotaxis, and TGF-β production^35,36^. Pf has been shown to possess anti-oxidative properties by reducing reactive free radicals shown in the rat liver cirrhosis model^37^ and mouse lung injury model^38^ as well as in the human pulmonary vascular smooth cells stimulated with sera of patients with idiopathic pulmonary^39^. Consistent with the findings in the literature, here we demonstrated that Pf at 0.5 mg/ml, significantly ameliorated neutrophil free radical production upon LPS activation, thus, supporting the existing evidence of Pf’s anti-oxidative activity and further strengthening its anti-inflammatory potential.

Neutrophils, when activated, along with free radical production, secrete a slew of pro-inflammatory cytokines, chemokines, and regulatory proteins to curtail infection and initiate the healing process^40^. These molecular events play a critical role in healing by mediating extracellular matrix production and tissue remodeling, epidermal-mesenchymal transition, and vasculogenesis^41^. High levels of TNFα, MCP-1, IL-6 and IL-8 are present in both burn and non-burn wound environments and their continued presence can worsen healing outcomes^42,43^. For examples, anti-chemokine treatment by anti-MCP-1 gene therapy has been shown to improve pulmonary fibrosis in mice^44^. Additionally, IL-1RA, an important natural anti-inflammatory protein, ameliorates murine and human cystic fibrosis, suggesting the importance of lowering inflammation for attaining better wound outcomes^45^. Furthermore, anti-inflammatory agents such as COX-2 (cyclooxygenase-2) inhibitors have been shown to reduce scar tissue formation in mouse full-thickness wounds^46^, whereas non-steroidal anti-inflammatory drugs may promote scar tissue formation^47^. In this context, Pf may be an ideal therapeutic for improving wound outcomes as it was shown to decrease inflammation in this study by reducing pro-inflammatory cytokines (TNFα, IL-1β & IL-6) and chemokines (IL-8, MCP-1 & MIP-1α) levels in LPS-activated neutrophils. It has also been previously shown that Pf possesses anti-fibrotic potential by reducing fibroblast proliferation and their transformation into myofibroblasts in vitro^48^. In our study though the effects including toxicity were concentration dependent, the drug action was more balanced with moderate loss to neutrophil antimicrobial functions at 0.5 mg/ml Pf concentration. Our data demonstrates that Pf exerts its anti-inflammatory activity by specifically modulating a number of neutrophil functions in inflammation.

Neutrophils store a plethora of proteins such as NGAL, defensins, lysozyme, MPO, MMP-8, MMP-9, and CD11b that participate in antimicrobial activity and tissue regeneration. These molecules are stored in several types of neutrophil granules such as azurophilic, secondary, tertiary, and specific granules^49^. The release of these granular proteins can be measured by the estimation of increase in specific cell surface protein expression^50^. Upon Pf treatment, we observed the decrease of these cell surface proteins on LPS-activated neutrophils suggesting that there were decreases in neutrophil degranulation. A similar reduction of a granular protein, MPO, was also observed in LPS-stimulated neutrophils treated with Pf and in our earlier study on the Pf treatment of mouse deep partial-thickness burn wounds^16^. Neutrophil degranulation proteins such as matrix metalloproteases increase tissue damage and promote fibrosis^51^. For these reasons, MMPs have been targeted for the treatment of idiopathic pulmonary fibrosis and other fibrotic conditions^52^. We observed that Pf decreased MMP-8 released from inflamed neutrophils, suggesting a broader effect of Pf in tissue preservation from the damaging consequences of various proteases. Conversely, the reduction in degranulation due to Pf treatment could negatively lessen the ability of neutrophils to kill extracellular bacteria to fight infection. Therefore, it is conceivable to include antimicrobials when considering topical Pf treatment to curtail prolonged inflammation of open skin wounds for improved outcomes such as reduced scarring.

Apart from antimicrobial degranulation, neutrophils fight infection by phagocytosis and NETosis which utilize cellular cytoskeleton machinery^41^. As kinases and MMPs (particularly MMP8^53^) are involved in triggering phagocytosis by re-organizing cellular cytoskeleton proteins, we observed that Pf treatment, which targets kinases^18,54^, resulted in a reduced phagocytic uptake of *Escherichia coli* bioparticle in LPS-stimulated neutrophils^55,56^. Furthermore, for host defense, neutrophils employ a unique suicidal mechanism, also driven by kinases, to trap microbes (NETosis) by releasing intracellular histones and DNA to form NETs^57^. While serving as defense units, these NETs molecules can collaterally act as DAMPs and exacerbate inflammation and are seen in excess in non-healing wounds and in fibrotic tissues^58^. Studies have shown that inhibition of NETosis resulted in accelerated healing^59^. Therefore, reduced neutrophil NETosis as observed after Pf treatment in our study suggests that the drug could improve healing outcomes.

LPS, which binds to TLR4, triggers downstream Myeloid differentiation primary response 88 (MYD88)-dependent and -independent signaling of mitogen-activated protein kinase (MAPK) and NF-κB pathways. The resulting oxidative burst and inflammatory response thereof, triggers the activation of multiple pathways such as Akt, extracellular-signal-regulated kinase (ERK) and JNK^60^. Combined, these signals result in neutrophil activation/hyper-activation and other related responses^61–63^. In line with these observations, the previous kinase analysis supports the activation of multiple kinases in neutrophils during inflammation and infection. In particular, the significant association of the p38MAPK, JNK-1/2/3, Akt-1/2/3, Src, PYK2, AMPKa-1/2, c-Jun, FAK and MSK-1/2 with neutrophil functions have been well studied^62–64^. We have previously shown that Pf decreases the pro-fibrotic phenotype of TGF-β-induced human fibroblasts by targeting the p38 MAPK^18^. In other studies, Pf also modulates Akt, Wnt/β-Catenin, and ERK pathway kinase proteins to prevent tumor cells proliferation^65^. Additionally, Pf reduced NLRP3 (Nucleotide-binding oligomerization domain, Leucine rich Repeat and Pyrin domain containing 3) and ASC (Apoptosis-associated speck-like protein containing a caspase recruitment domain) pathway protein expression to ameliorate LPS-induced pulmonary inflammation^38^. Collectively, depending on cell types, the evidence suggested that Pf might target different kinases and proteins. Consistent with earlier studies on various cell types, Pf treatment also affected multiple pathway kinases and proteins in LPS-stimulated neutrophils 3 h post treatment. However, due to the broad action of Pf and the plethora of functional attributes neutrophils possess, it is very challenging based on our present data to pinpoint the Pf treatment targets that lead to phenotypic changes observed in these LPS-activated neutrophils. It is plausible that we might have to examine the earlier time points post stimulation to gain more insights on specific drug targets for the resultant phenotypic alterations observed in these cells in future studies.

Lastly, to the best of our knowledge, using a multitude of different in vitro assays, this is the first study to demonstrate the anti-inflammatory effects of Pf against a wide-range of inflammatory properties displayed by LPS-stimulated neutrophils, hence strengthening the anti-inflammatory potential of this therapeutic agent. However, the limitation of the present study is that this is an in vitro mimetic model which lacks the crosstalk between multiple immune cell types. Evaluation of drug interactions with innate immune cells in in vivo models of pathology will help to broaden the understanding of Pf as an anti-inflammatory agent. This could potentially lead to repurposing the drug for treatment of other pathologies involving underlying chronic inflammation.

## Methods

### Cells

Fresh blood in EDTA vacutainer tubes from healthy donors was kindly provided by the Blood Collection Center for Research, USAISR Donor Center. Neutrophils were isolated within 1 h of blood draw using the MACSxpress whole blood (human) neutrophil isolation kit (Miltenyi Biotec, Auburn, CA) and red blood cell lysis was performed using erythrocyte depletion kit (Miltenyi Biotec) per manufacturer’s instructions. Isolated neutrophils were washed once with Hank’s balanced salt solution without Ca^2+^ and Mg^2+^ [HBSS] (Gibco-Life Technologies, Carlsbad, CA, USA) and suspended in media containing RPMI1640 (ATCC, Manassas, VA, USA) supplemented with 0.5% human serum albumin (Celprogen Inc., Torrance, CA, USA)^66^ and 1% penicillin–streptomycin (Gibco-Life Technologies). Cell viability was determined by trypan blue exclusion method using a TC20 automated cell counter (Bio-Rad Laboratories Inc., Hercules, CA, USA). After staining the cells with the CD15 & CD16 antibodies (Miltenyi Biotec) per manufacturer’s instructions, neutrophil purity (Supplementary Figure S1; mean ± SD is 96.6 ± 1.6) was tested using the Attune NxT flow cytometer (Invitrogen-Life Technologies, Carlsbad, CA USA), Majority of the experiments/assays were performed with blood from different donors on different days.

### Treatments

Well plates/flasks were coated with 50 µg/ml of Matrigel (Corning Incorporated Life Sciences, Durham, NC, USA) per manufacturer’s instructions and were used in the study. Due to increasing evidences suggesting the importance of 3D cell culture and immune cell-tissue interactions^67–69^, Matrigel was used to mimic tissue micro-environment. Appropriate densities of freshly isolated neutrophils were plated into the wells of the Matrigel coated flasks/well plate and incubated for 30 min at room temperature to let neutrophils attach to the wells. The plates/flasks were placed in a CO_2_ incubator set at 37 °C until further use.

Neutrophils (30,000/well of 96 well plate; unless stated otherwise) were supplemented first with 0 to 1 mg/ml of Pf (AK Scientific, Union City, CA, USA), followed by 0 (mock) or 20 ng/ml (LPS) of LPS-O111 (Sigma, St. Louis, MO, USA). For NETosis assay, 0 or 40 µg/ml of *E. coli* LPS-O128 (Sigma) was used instead. All reagents were prepared in pre-warmed media.

### Apoptosis and toxicity assay

Assay was carried out in Incucyte S3 live cell imaging system at 37 °C with 5% CO_2_ (Essen Biosciences Inc., Ann Arbor, MI). Briefly, neutrophils (± Pf ± LPS) were treated with 20 µl of recommended concentration of Cytotox red reagent (Essen Biosciences Inc.), gently mixed, and live-bright field/fluorescent images were recorded at various intervals for up to 16 h using a 4 × objective in the Incucyte S3 system which was effectively used by other groups to understand the neutrophil behavior including apoptosis/toxicity/chemotaxis/NETosis^70,71^. Image analysis was done using the software provided (IncucyteS3 Software; Essen Biosciences Inc.). Percent dead cells were graphed from red fluorescent cell count relative to total cell count. 1% hydrogen peroxide was used as a positive control for toxicity assay. In another assay, live-images (Fig. 1a) from neutrophils (± Pf ± LPS), supplemented with 20 µl of recommended concentration of Annexin-V green and Caspase-3/7 red (Essen Biosciences Inc.), were used to estimate apoptosis instead. Percent apoptotic cells were calculated using the sum total of Annexin-V, Caspase-3/7, and double positive cells in relation to the total cell number. Five µM staurosporine (ssp) was used as a positive control for apoptosis assay.

### Chemotaxis assay

The Incucyte live cell imaging system (Essen Biosciences Inc.) was used to study chemotaxis^66^. Chemotaxis of neutrophils (± Pf ± LPS) was assayed for up to 12 h in Clearview 96-well chemotaxis plates (Essen Biosciences Inc.) with fMLP (300 nM; Sigma), LTB4 (100 nM; Tocris Bioscience), IL-8 (100 ng/ml; R&D Bio), or TGF-β (100 ng/ml; R&D Bio) as chemo-attractants according to manufacturer’s instructions. Number of cells migrated to the bottom-side of the trans-well in the time interval was measured for all treatment groups based on the live-images obtained using the software provided with the system (Fig. 2a). The measurements were normalized to the cell number on the top-side of the trans-well at time 0. The measurements are not additive over time as the cells which settled in the bottom well that moved down from the bottom side of trans-well may not be counted. Random migration measured from neutrophils migrating to media alone was discounted from all test conditions. 1 µM cyt-D was used as a negative control. Results were graphed as the number of migrated cells over time as the measurement of neutrophil chemotaxis.

### Neutrophil oxidative burst

Reactive oxygen and nitrogen species were measured using free radical sensors from Life Technologies—CellROX Green (intracellular ROS) and OxyBURST Green H2HFF BSA (extracellular/secreted ROS) and DAF-FM Diacetate (total nitric oxide; NO). Neutrophils (± Pf ± LPS) were supplemented with recommended concentrations of CellROX Green or DAF-FM Diacetate. Live images for intracellular ROS (Fig. 3a) or mean fluorescent intensities of wells for NO estimation were recorded for up to 2 h in an Incucyte S3 live cell imaging system. Intracellular ROS intensity and total NO intensity for different conditions with appropriate background subtraction was calculated and plotted using the instrument software.

For measuring extracellular ROS, 2 × 10^5^ neutrophils (± Pf ± LPS) were supplemented with the recommended concentration of OxyBURST Green H2HFF BSA. Fluorescent intensity readings [@490/520 nm (ex/em)] were recorded immediately at 37 °C for up to 15 min using a Cytation 5 cell imaging reader w/ Gen5.303 BioTek microplate reader and imager software (BioTek Instruments Inc.; USA). Blanked data was used to estimate the oxidative burst of neutrophils.

### Degranulation and pro-inflammatory response

Neutrophils (± Pf ± LPS) at 1 × 10^6^ cells/500 µl/well in 12 well plates were incubated at 37 °C for 4 and 16 h. Cells were spun down, supernatants were collected and stored at − 80 °C. Plates were washed with cold HBSS and cells were gently lifted off with a cell scraper, spun down, and resuspended in 100 µl DPBS containing 0.5% HSA. Staining was carried out with CD11b, CD35, CD45 and CD63 or CD14, CD15, CD16, and CD66b fluorescent antibodies (Miltenyi Biotec) per manufacturer’s instructions. Fluorescent events and intensities were recorded by flow cytometry w/ an auto-sampler using Attune NxT software (Attune; Life Technologies). Percentage fluorescent cells and mean fluorescent intensity (MFI) were measured after gating was performed with isotype controls, appropriate FMO (Fluorescence minus One), compensation, and unstained controls for 50,000 total events and a minimum of 10,000 gated events (Propidium Iodide negative).

Supernatants were assayed for degranulation and inflammation markers such as MMP-8, MMP-9, NGAL, cytokines (TNFα, IL-1β, and IL-6), chemokines (IL-8, MCP-1, MIP-1α, and MIP-1β) and protein regulators (Granulocyte–macrophage colony-stimulating factor/GM-CSF, IL-10, and IL-1RA) using the Procarta 13-plex assay kit (ThermoFisher Scientific; USA). Assay was performed according to manufacturer’s instructions and quantification was done using a Bioplex 200 system w/ Bioplex manager software v6.1 (Bio-Rad Laboratories, Inc.; USA). Data was plotted with absolute quantity of protein from 6 donors. Myeloperoxidase (MPO) in supernatants was measured according to manufacturer’s instructions after the assay procedure was performed with the FLUOROMPO assay kit (CellPro Technologies). Fluorescence measurements were made using a Cytation-5 fluorescence reader (BioTek) and data was plotted as MFI of MPO from 6 donors.

### Phagocytosis assay

Neutrophil (± Pf ± LPS) phagocytosis of pHrodo green *E. coli* Bioparticles (Essen Biosciences Inc.) was measured over a period of 12 h using the Incucyte S3 Live Cell Imaging System per manufacturer’s instructions. One µM cyto-D was used as a negative control. The reagent utilizes the principle of non-fluorescent *E. coli* Bioparticles turning fluorescent (Fig. 6a) upon acidification after phagocytosis. Image analysis was done using the software provided with the system. Percent fluorescent cells and normalized mean fluorescence intensity were graphed from data collected.

### NETosis

For measuring NETosis, neutrophils (± Pf ± LPS) were incubated for 4 h in an incubator set at 37 °C with 5% CO_2_. Post incubation, plates were fixed with 4% para-formaldehyde and stained (w/o permeabilization of cell membranes) with anti-human elastase primary antibody (R&D Bio), followed by the corresponding Alexa-fluor secondary antibody (R&D Bio) and Hoechst 33342 for nuclear staining (BD) based on manufacturer’s instructions. Post staining, microscopic images were taken at 10 × magnification using a Cytation-5 cell imaging reader. Number of cells forming NETs (total cell number minus non-NETosis cell number), based on average nucleus size was calculated. Phorbol myristate acetate (PMA; 25 nM) was used as a positive control. Fluorescent intensities from anti-neutrophil elastase antibody staining (with the appropriate background subtraction) were measured using the instrument software. Because the cells were not permeabilized, therefore only the elastase-component of NETs was stained.

### Kinase signaling pathway array

Neutrophils (± LPS) with 0 or 0.5 mg/ml Pf (10^7^ cells/5 ml) were incubated for 3 h in a 5% CO_2_ incubator set at 37 °C. Post incubation cells were harvested, lysed using a lysis buffer supplemented with Halt protease and phosphatase inhibitor cocktail (ThermoScientific, Rockford, IL, USA), and assayed per instructions of the Proteome Profiler antibody arrays (R&D Systems, Inc., Minneapolis, MN, USA). The phosphorylated protein content (post staining) of the array was determined by the chemiluminescent reaction and quantified by densitometry by Chemidoc touch imaging system using the Image Lab software (v5.2.1; Bio-Rad Laboratories, Inc., Hercules, CA, USA). The phosphorylated protein content was normalized to the total protein content (determined by Pierce BCA protein assay kit) per test condition with an appropriate background subtraction. Mean intensity values from one representative donor were plotted as heat map. The graph with the phosphorylated protein quantity was plotted with the mean and SEM of data from 6 donors. The repetitive presentation of a single protein in the data plots represents different phosphorylation sites of that particular protein.

### Statistical analysis

For all time course experiments, time was given relative to LPS/Pf treatment. Data was plotted and analyzed using the Graphpad Prism v7 (GraphPad Software, San Diego, CA, USA) for individual time points and within donors. Statistical significance for all the tests (for each particular time point) was determined by the two-way ANOVA with repeated measures using the Bonferroni post-hoc analysis. Statistical significance, *p* ≤ 0.05, was indicated by the asterisk (*).

## Supplementary information

Supplementary Information.

## References

[1] Nathan C (2006). Neutrophils and immunity: challenges and opportunities. Nat. Rev. Immunol..

[2] Rosales C (2018). Neutrophil: a cell with many roles in inflammation or several cell types?. Front. Physiol..

[3] Weiss SJ (1989). Tissue destruction by neutrophils. N. Engl. J. Med..

[4] Dovi JV, Szpaderska AM, DiPietro LA (2004). Neutrophil function in the healing wound: adding insult to injury?. Thromb. Haemost..

[5] Newton K, Dixit VM (2012). Signaling in innate immunity and inflammation. Cold Spring Harb. Perspect. Biol..

[6] Wilgus TA, Roy S, McDaniel JC (2013). Neutrophils and wound repair: positive actions and negative reactions. Adv. Wound Care (New Rochelle).

[7] Mortaz E, Alipoor SD, Adcock IM, Mumby S, Koenderman L (2018). Update on neutrophil function in severe inflammation. Front. Immunol..

[8] Leliefeld PH, Wessels CM, Leenen LP, Koenderman L, Pillay J (2016). The role of neutrophils in immune dysfunction during severe inflammation. Crit. Care.

[9] Ogawa R (2017). Keloid and hypertrophic scars are the result of chronic inflammation in the reticular dermis. Int. J. Mol. Sci..

[10] Martin P (2003). Wound healing in the PU.1 null mouse—tissue repair is not dependent on inflammatory cells. Curr. Biol..

[11] Dovi JV, He LK, DiPietro LA (2003). Accelerated wound closure in neutrophil-depleted mice. J. Leukoc. Biol..

[12] Noble PW (2016). Pirfenidone for idiopathic pulmonary fibrosis: analysis of pooled data from three multinational phase 3 trials. Eur. Respir. J..

[13] Noble PW (2011). Pirfenidone in patients with idiopathic pulmonary fibrosis (CAPACITY): two randomised trials. Lancet.

[14] Dorati R, Medina JL, DeLuca PP, Leung KP (2018). Development of a topical 48-h release formulation as an anti-scarring treatment for deep partial-thickness burns. AAPS PharmSciTech.

[15] Wells AR, Leung KP (2020). Pirfenidone attenuates the profibrotic contractile phenotype of differentiated human dermal myofibroblasts. Biochem. Biophys. Res. Commun..

[16] Medina JL, Sebastian EA, Fourcaudot AB, Dorati R, Leung KP (2019). Pirfenidone ointment modulates the burn wound bed in C57BL/6 mice by suppressing inflammatory responses. Inflammation.

[17] Takeda Y, Tsujino K, Kijima T, Kumanogoh A (2014). Efficacy and safety of pirfenidone for idiopathic pulmonary fibrosis. Patient Prefer. Adherence.

[18] Hall CL, Wells AR, Leung KP (2018). Pirfenidone reduces profibrotic responses in human dermal myofibroblasts, in vitro. Lab. Investig..

[19] Knuppel L (2017). A novel antifibrotic mechanism of nintedanib and pirfenidone. Inhibition of collagen fibril assembly. Am. J. Respir. Cell Mol. Biol..

[20] Toda M (2018). Pirfenidone suppresses polarization to M2 phenotype macrophages and the fibrogenic activity of rat lung fibroblasts. J. Clin. Biochem. Nutr..

[21] Liu X (2017). The antiangiogenesis effect of pirfenidone in wound healing in vitro. J. Ocul. Pharmacol. Ther..

[22] Kruger P (2015). Neutrophils: between host defence, immune modulation, and tissue injury. PLoS Pathog..

[23] Ngkelo A, Meja K, Yeadon M, Adcock I, Kirkham PA (2012). LPS induced inflammatory responses in human peripheral blood mononuclear cells is mediated through NOX4 and Gialpha dependent PI-3kinase signalling. J. Inflamm. (Lond.).

[24] Park BS, Lee JO (2013). Recognition of lipopolysaccharide pattern by TLR4 complexes. Exp. Mol. Med..

[25] Hidalgo A, Chilvers ER, Summers C, Koenderman L (2019). The neutrophil life cycle. Trends Immunol..

[26] Lancaster LH (2017). Pirfenidone safety and adverse event management in idiopathic pulmonary fibrosis. Eur. Respir. Rev..

[27] Dosanjh A (2006). Pirfenidone: anti-fibrotic agent with a potential therapeutic role in the management of transplantation patients. Eur. J. Pharmacol..

[28] Nathan SD (2018). Dose modification and dose intensity during treatment with pirfenidone: analysis of pooled data from three multinational phase III trials. BMJ Open Respir. Res..

[29] Lin X, Yu M, Wu K, Yuan H, Zhong H (2009). Effects of pirfenidone on proliferation, migration, and collagen contraction of human Tenon's fibroblasts in vitro. Investig. Ophthalmol. Vis. Sci..

[30] Didiasova M (2017). Pirfenidone exerts antifibrotic effects through inhibition of GLI transcription factors. FASEB J..

[31] Baggiolini M, Loetscher P, Moser B (1995). Interleukin-8 and the chemokine family. Int. J. Immunopharmacol..

[32] de Oliveira S, Rosowski EE, Huttenlocher A (2016). Neutrophil migration in infection and wound repair: going forward in reverse. Nat. Rev. Immunol..

[33] van de Goot F (2009). Acute inflammation is persistent locally in burn wounds: a pivotal role for complement and C-reactive protein. J. Burn Care Res..

[34] Jones HA, Schofield JB, Krausz T, Boobis AR, Haslett C (1998). Pulmonary fibrosis correlates with duration of tissue neutrophil activation. Am. J. Respir. Crit. Care Med..

[35] Shroff A, Mamalis A, Jagdeo J (2014). Oxidative stress and skin fibrosis. Curr. Pathobiol. Rep..

[36] Yoo SK, Starnes TW, Deng Q, Huttenlocher A (2011). Lyn is a redox sensor that mediates leukocyte wound attraction in vivo. Nature.

[37] Salazar-Montes A, Ruiz-Corro L, Lopez-Reyes A, Castrejon-Gomez E, Armendariz-Borunda J (2008). Potent antioxidant role of pirfenidone in experimental cirrhosis. Eur. J. Pharmacol..

[38] Pourgholamhossein F (2018). Pirfenidone protects against paraquat-induced lung injury and fibrosis in mice by modulation of inflammation, oxidative stress, and gene expression. Food Chem. Toxicol..

[39] Fois AG (2018). Antioxidant activity mediates pirfenidone antifibrotic effects in human pulmonary vascular smooth muscle cells exposed to sera of idiopathic pulmonary fibrosis patients. Oxid. Med. Cell Longev..

[40] Reinke JM, Sorg H (2012). Wound repair and regeneration. Eur. Surg. Res..

[41] Wright HL, Moots RJ, Bucknall RC, Edwards SW (2010). Neutrophil function in inflammation and inflammatory diseases. Rheumatology (Oxford).

[42] Farina JA, Rosique MJ, Rosique RG (2013). Curbing inflammation in burn patients. Int. J. Inflamm..

[43] Wynn TA (2008). Cellular and molecular mechanisms of fibrosis. J. Pathol..

[44] Inoshima I (2004). Anti-monocyte chemoattractant protein-1 gene therapy attenuates pulmonary fibrosis in mice. Am. J. Physiol. Lung Cell Mol. Physiol..

[45] Iannitti RG (2016). IL-1 receptor antagonist ameliorates inflammasome-dependent inflammation in murine and human cystic fibrosis. Nat. Commun..

[46] Wilgus TA, Vodovotz Y, Vittadini E, Clubbs EA, Oberyszyn TM (2003). Reduction of scar formation in full-thickness wounds with topical celecoxib treatment. Wound Repair Regen..

[47] Su WH (2010). Nonsteroidal anti-inflammatory drugs for wounds: pain relief or excessive scar formation?. Mediat. Inflamm..

[48] Spond J (2003). Inhibition of experimental acute pulmonary inflammation by pirfenidone. Pulm. Pharmacol. Ther..

[49] Lacy P (2006). Mechanisms of degranulation in neutrophils. Allergy Asthma Clin. Immunol..

[50] Naegelen I (2015). Regulation of neutrophil degranulation and cytokine secretion: a novel model approach based on linear fitting. J. Immunol. Res..

[51] Craig VJ (2013). Profibrotic activities for matrix metalloproteinase-8 during bleomycin-mediated lung injury. J. Immunol..

[52] Craig VJ, Zhang L, Hagood JS, Owen CA (2015). Matrix metalloproteinases as therapeutic targets for idiopathic pulmonary fibrosis. Am. J. Respir. Cell Mol. Biol..

[53] Tester AM (2007). LPS responsiveness and neutrophil chemotaxis in vivo require PMN MMP-8 activity. PLoS ONE.

[54] Korfei M (2018). Comparison of the antifibrotic effects of the pan-histone deacetylase-inhibitor panobinostat versus the IPF-drug pirfenidone in fibroblasts from patients with idiopathic pulmonary fibrosis. PLoS ONE.

[55] Shi Y (2006). Protein-tyrosine kinase Syk is required for pathogen engulfment in complement-mediated phagocytosis. Blood.

[56] Nordenfelt P, Tapper H (2011). Phagosome dynamics during phagocytosis by neutrophils. J. Leukoc. Biol..

[57] Yipp BG, Kubes P (2013). NETosis: how vital is it?. Blood.

[58] Law SM, Gray RD (2017). Neutrophil extracellular traps and the dysfunctional innate immune response of cystic fibrosis lung disease: a review. J. Inflamm. (Lond.).

[59] Fadini GP (2016). NETosis delays diabetic wound healing in mice and humans. Diabetes.

[60] Kato T, Kitagawa S (2006). Regulation of neutrophil functions by proinflammatory cytokines. Int. J. Hematol..

[61] Mocsai A, Walzog B, Lowell CA (2015). Intracellular signalling during neutrophil recruitment. Cardiovasc. Res..

[62] Azcutia V, Parkos CA, Brazil JC (2017). Role of negative regulation of immune signaling pathways in neutrophil function. J. Leukoc. Biol..

[63] Diks SH (2004). Kinome profiling for studying lipopolysaccharide signal transduction in human peripheral blood mononuclear cells. J. Biol. Chem..

[64] Naccache PH (2013). Signalling in neutrophils: a retro look. ISRN Physiol..

[65] Li C (2018). Pirfenidone decreases mesothelioma cell proliferation and migration via inhibition of ERK and AKT and regulates mesothelioma tumor microenvironment in vivo. Sci. Rep..

[66] Bevan N (2019). Real-time visualization and quantification of neutrophil activation and function using live-cell analysis. J. Immunol..

[67] Rebelo SP (2018). 3D–3-culture: a tool to unveil macrophage plasticity in the tumour microenvironment. Biomaterials.

[68] Evani SJ, Dallo SF, Ramasubramanian AK (2016). Biophysical and biochemical outcomes of *Chlamydia**pneumoniae* infection promotes pro-atherogenic matrix microenvironment. Front. Microbiol..

[69] Gonzalez-Simon AL, West JL, McIntire LV, Smith CW (2006). ECM interactions with neutrophil integrins regulate cell activity: an engineering approach to studying cell motility in inflammation. FASEB J..

[70] Gupta S, Chan DW, Zaal KJ, Kaplan MJ (2018). A high-throughput real-time imaging technique to quantify NETosis and distinguish mechanisms of cell death in human neutrophils. J. Immunol..

[71] Blez D (2020). Ibrutinib induces multiple functional defects in the neutrophil response against *Aspergillus fumigatus*. Haematologica.

