# Supplementary material for: Pirfenidone regulates LPS mediated activation of neutrophils
Source: Sci Rep. 2020 Nov 17;10:19936. doi: 10.1038/s41598-020-76271-3 (PMC7672086; doi:10.1038/s41598-020-76271-3)
Supplement: Supplementary file 1 — Supplementary Information. [file 41598_2020_76271_MOESM1_ESM.pdf]

PIRFENIDONE REGULATES LPS MEDIATED ACTIVATION OF NEUTROPHILS

Shankar J Evani[1], S L Rajasekhar Karna[1], Seshu J[2], Leung KP[1,*]

[1] Division of Combat Wound Repair, US Army Institute of Surgical Research, JBSA Fort Sam Houston, TX, USA
[2] South Texas Center for Emerging Infectious Diseases (STCEID) and Department of Biology, The University of Texas at San Antonio, One UTSA Circle, San Antonio, TX, USA
[*] Corresponding author

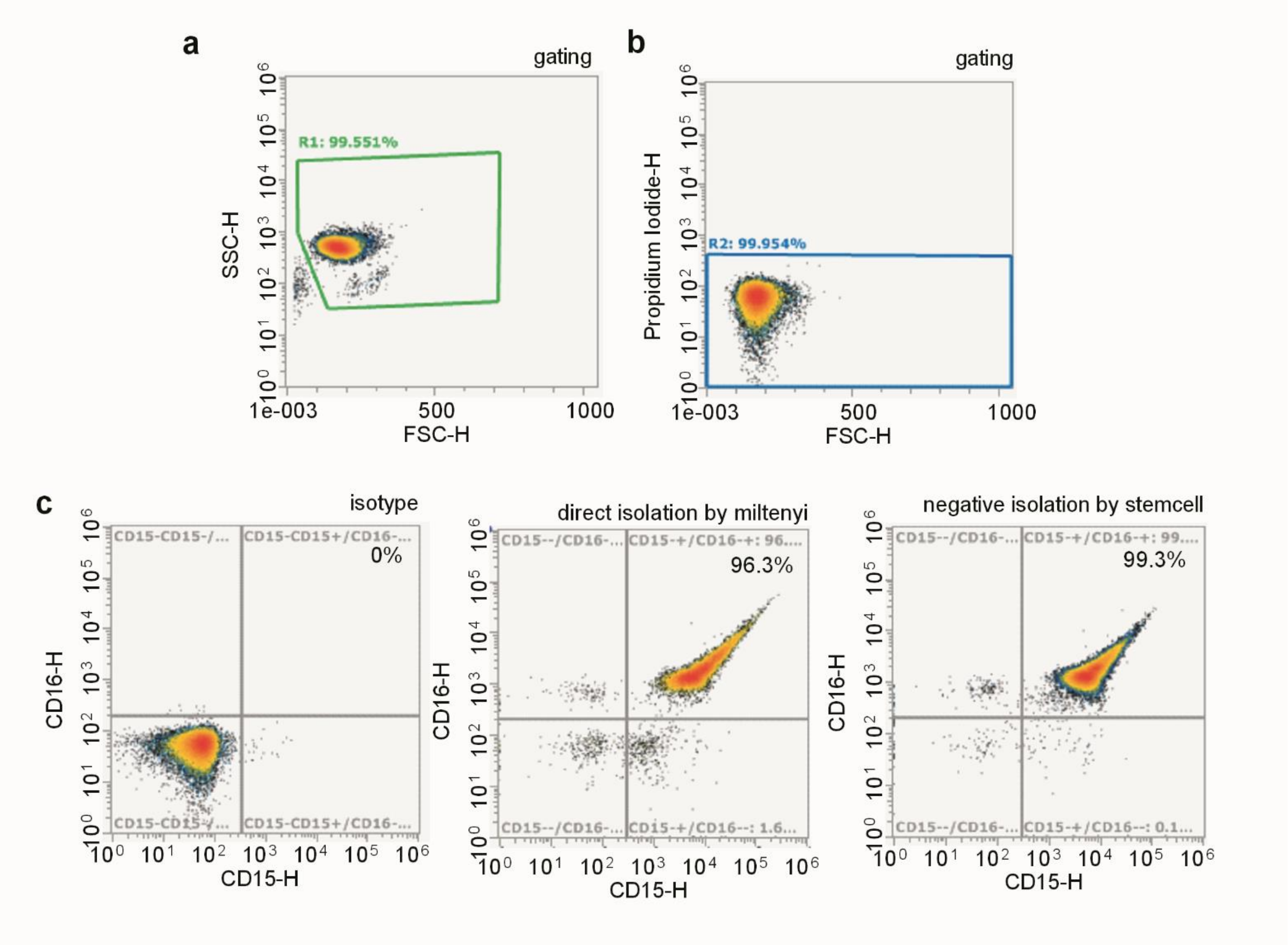

Figure S1. Neutrophil isolation and purity determination: Neutrophils were isolated within 1 hour of blood draw using various methods. Isolated neutrophils were tested for purity (CD15+/CD16+) using the Attune NxT flow cytometer after staining with respective antibodies. Debris and dead cells were eliminated by excluding low SSC/FSC events (a) and Propidium Iodide negative cell population-gate R2 (b) respectively. Density plots with isotype controls, neutrophils isolated by MACSxpress whole blood human neutrophil isolation kit (96.6±1.6; mean±SEM) and Stem Cell negative isolation kit (99.43±0.15; mean±SEM) showing gates with CD15+/CD16+ (upper right quadrant) events as determinant of purity (c).

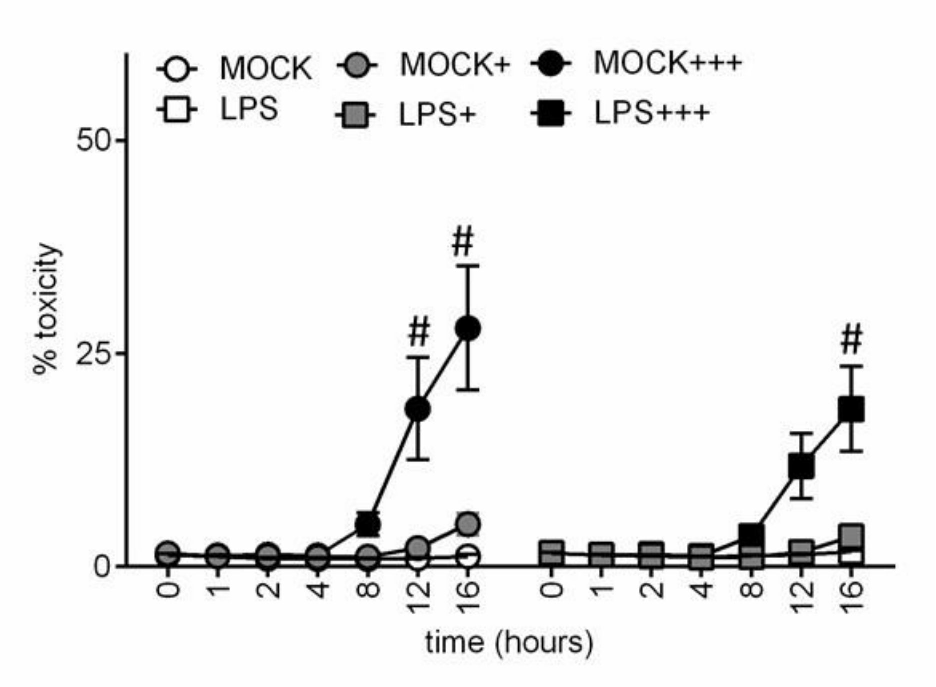

Figure S2. Effect of Pf on neutrophil cell health. Neutrophils in Matrigel-coated plates were treated with 0 ng/ml LPS + [Pf @ 0 (Mock) or 0.1 (Mock+) or 1(Mock+++) mg/ml] or 20 ng/ml LPS + [Pf @ 0 (LPS) or 0.1 (LPS+) and 1(LPS+++) mg/ml] along Cytotox-Red dye. Toxicity was measured by live cell fluorescent imaging of events using Incucyte S3 system. Graph showing percentage positive cells for Cytotox representing toxicity was plotted. Data represents mean ± SEM values from 6 donors. #represent significant difference with p value < 0.05 (Two-way ANOVA) between Pf treated *vs*. untreated neutrophils within mock or LPS groups.

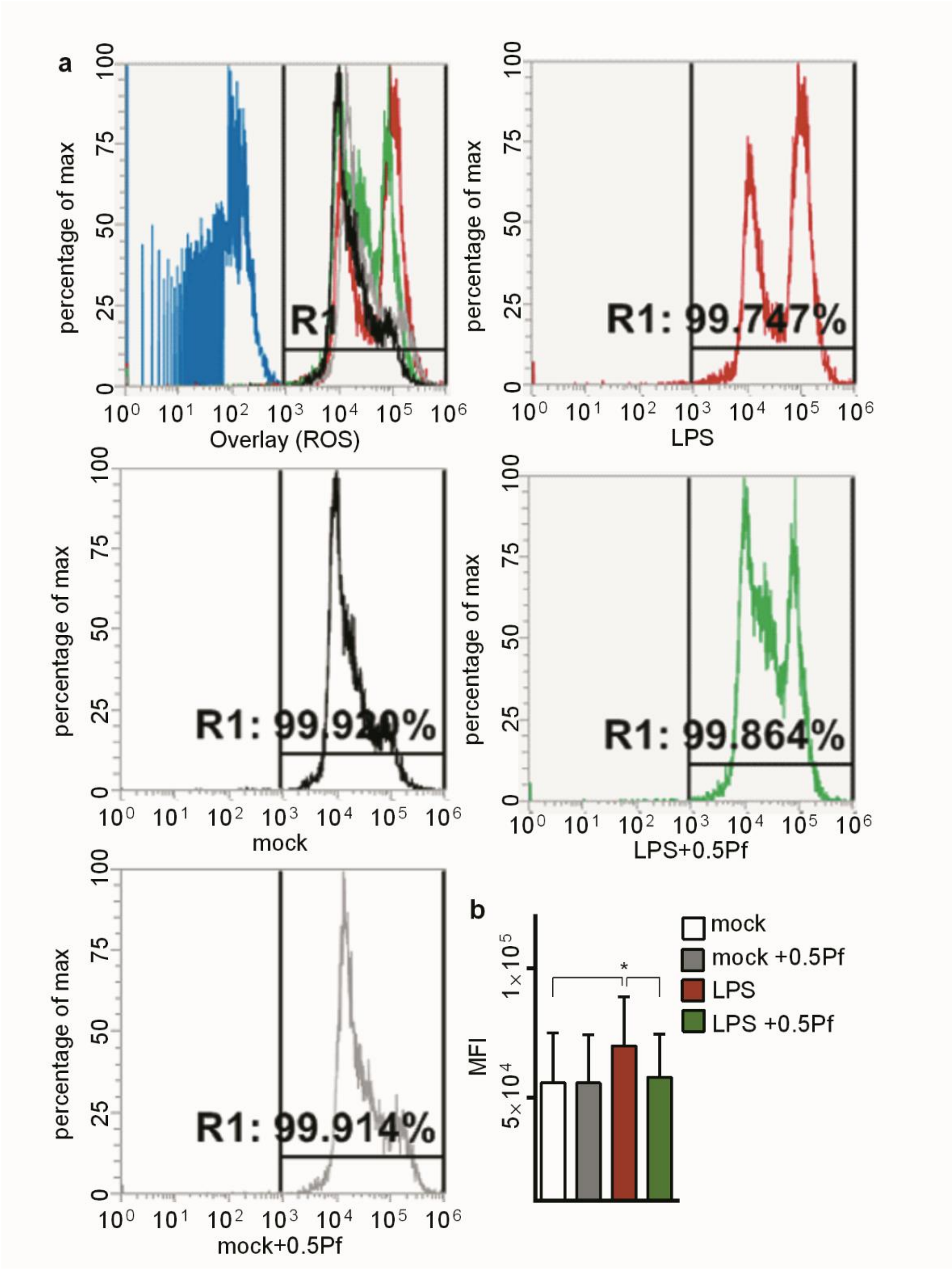

Figure S3. Neutrophil ROS production by flow cytometry. CellROX Green (intracellular ROS) dye was used to measure free radical production in neutrophils by flow cytometry at 1hour post treatment with Pf and LPS. (a) Histograms with R1 (ROS positive gate) for various conditions are shown. (b) Mean fluorescent intensity (MFI) of ROS from 4 donors were calculated, graphed (mean ± SD) and each experiment was performed in duplicates. * represents significant difference (p-value ≤ 0.05; two-way ANOVA) between various treatment groups.

Table S1. Effect of Pf on neutrophil functions.

4 h (mean ±SEM; # p < 0.05)	MOCK +	MOCK +++	LPS +	LPS +++
(_pg/ml)				
TNFα	24.6±2.5	23.3±2.4	47.6±8.9; #	32.9±9.1; #
IL-1β	17±2.7	18.7±3.5	20.7±6.9; #	78.7±32.4
IL-6	35±7.3	28.2±6	309.1±136.2	80.4±17.1; #
IL-1RA	35.1±2.6	30.7±5.1	247±64.1	123.8±36.4
MCP-1	17±2.7	14.6±1.7	17.5±3.8; #	23±9.8
IL-8	260.7±47.4	114.4±32.7; #	1922±431	397.4±118.2; #
MIP-1α	98.7±18	45.6±4.6; #	1687±271.5	327.6±78.6; #
MIP-1β	709.6±102.4	263.5±58.6; #	5284±253.6	1969±516.6; #

Neutrophils in Matrigel-coated plates were treated with 0 ng/ml LPS + [Pf @ 0.1 (Mock+) or 1(Mock+++) mg/ml] or 20 ng/ml LPS + [Pf @ 0.1 (LPS+) and 1(LPS+++) mg/ml] and incubated for 4 hours at 37ºC. Supernatants were collected and inflammation was measured by the Procarta multiplex assay. Data represents mean ± SEM values from 6 donors. # represent significant difference with a p value < 0.05 (Two-way ANOVA) between Pf treated *vs*. untreated neutrophils within mock or LPS groups.

Table S2. Effect of Pf on neutrophil functions.

16 h (mean ±SEM; # p < 0.05)	MOCK	MOCK ++	LPS	LPS ++
(_pg/ml)				
TNFα	29.1±1.4	25±5.3	76.5±11.3; *	28.6±3.2; #
IL-1β	13.7±2	13.2±3.5	28.3±9.2	26±4.6
IL-6	33±9.3	29.5±6.2	1407±509.6; *	1345±485.9
IL-1RA	63±4.1	54±11.1	255.8±40.7; *	535.4±95.8; #
MCP-1	15.8±1.4	14.4±3.2	35.4±8.3; *	13.4±1.4; #
IL-8	925±103.1	349.2±20.9; #	6553±1731.5; *	2703±346.3; #
MIP-1α	137.5±26.8	66.9±10.9; #	4352±0352.2; *	1933±345; #
MIP-1β	1104±258.1	634.7±48.9; #	5800±814.8; *	5130±281

Neutrophils in Matrigel-coated plates were treated with 0 ng/ml LPS + [Pf @ 0 (Mock) or 0.5 (Mock++) mg/ml] or 20 ng/ml LPS + [Pf @ 0 (LPS) or 0.5 (LPS++) mg/ml] and incubated for 16 hours at 37ºC. Supernatants were collected and inflammation was measured by the Procarta multiplex assay. Data represents mean ± SEM values from 6 donors. * represent significant difference with p value < 0.05 (Two-way ANOVA) between mock *vs*. LPS treated neutrophils. # represent significant difference with p value < 0.05 (Two-way ANOVA) between Pf treated *vs*. untreated neutrophils within mock or LPS groups.

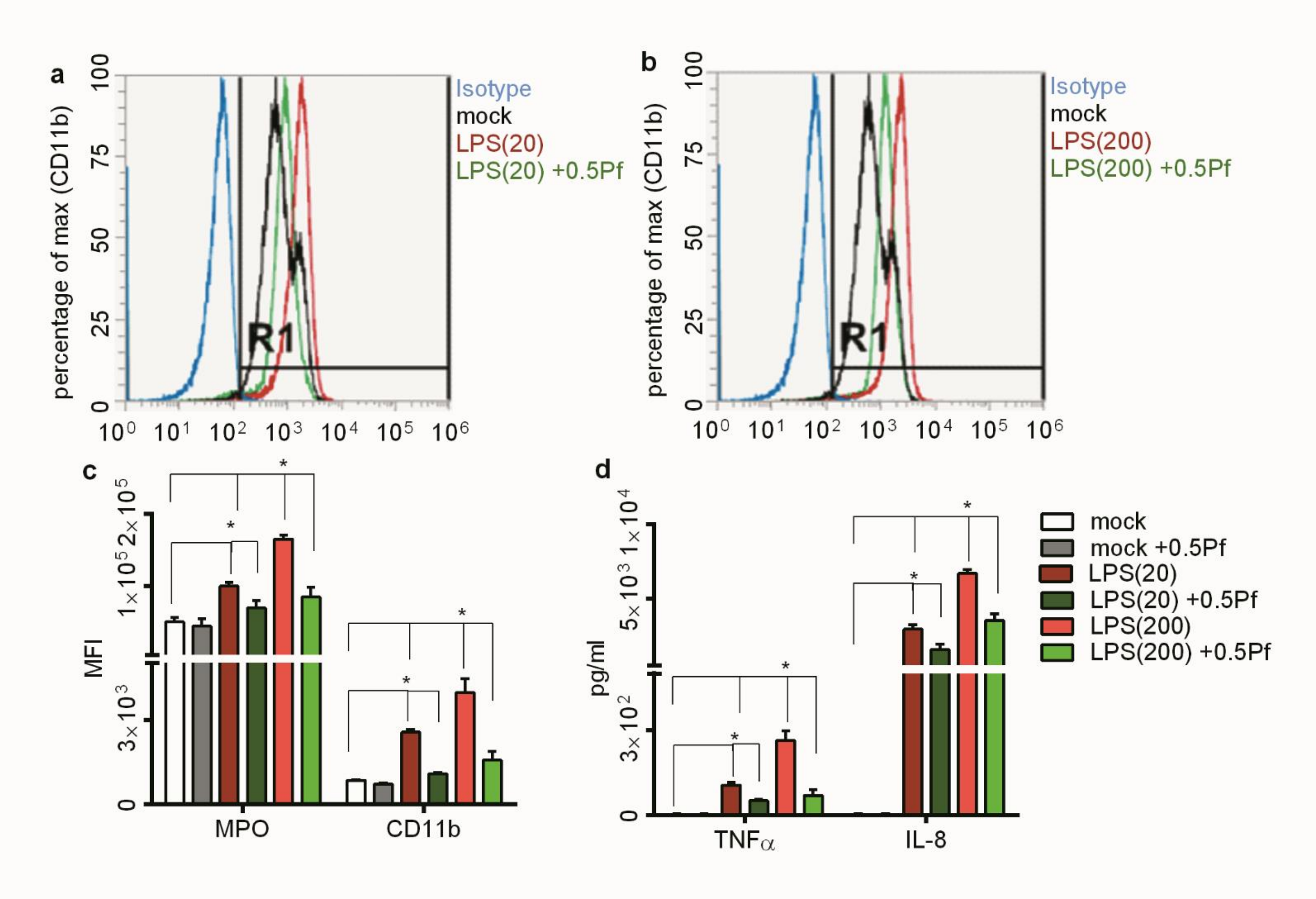

Figure S4. Effect of neutrophil purity and LPS concentration on drug action. Neutrophils were isolated within 1 hour of blood draw using the stem cell negative isolation per the manuscript by Federica Calzetti,, *et al*, 2016 [1]. Neutrophils thus obtained were treated with Pf at 0 or 0.5 mg/ml final concentration and activated with LPS (0 or 20 or 200 ng/ml). Cells were incubated for 4 hours at 37ºC. Cells and supernatants were collected for further analysis. Cells were stained with CD11b antibody to measure degranulation by flow cytometry. Supernatants were analyzed for MPO by fluorimetry using Cytation5. The supernatants were also assayed for TNFα & IL-8 (R&D Systems; USA) by DSX automated ELISA system w/ Revelation DSX Software (Dynex technologies; USA). The histograms of CD11b positive neutrophils with appropriate isotype controls are represented here (a, b). Bar graphs representing expression levels of (c) MPO-MFI, CD11b-MFI and (d) pg/ml of TNFα & IL-8 by neutrophils as mean ± SD values from 3 donors are shown. * represents significant difference (p-value $\leq$ 0.05; two-way ANOVA) between various treatment groups.

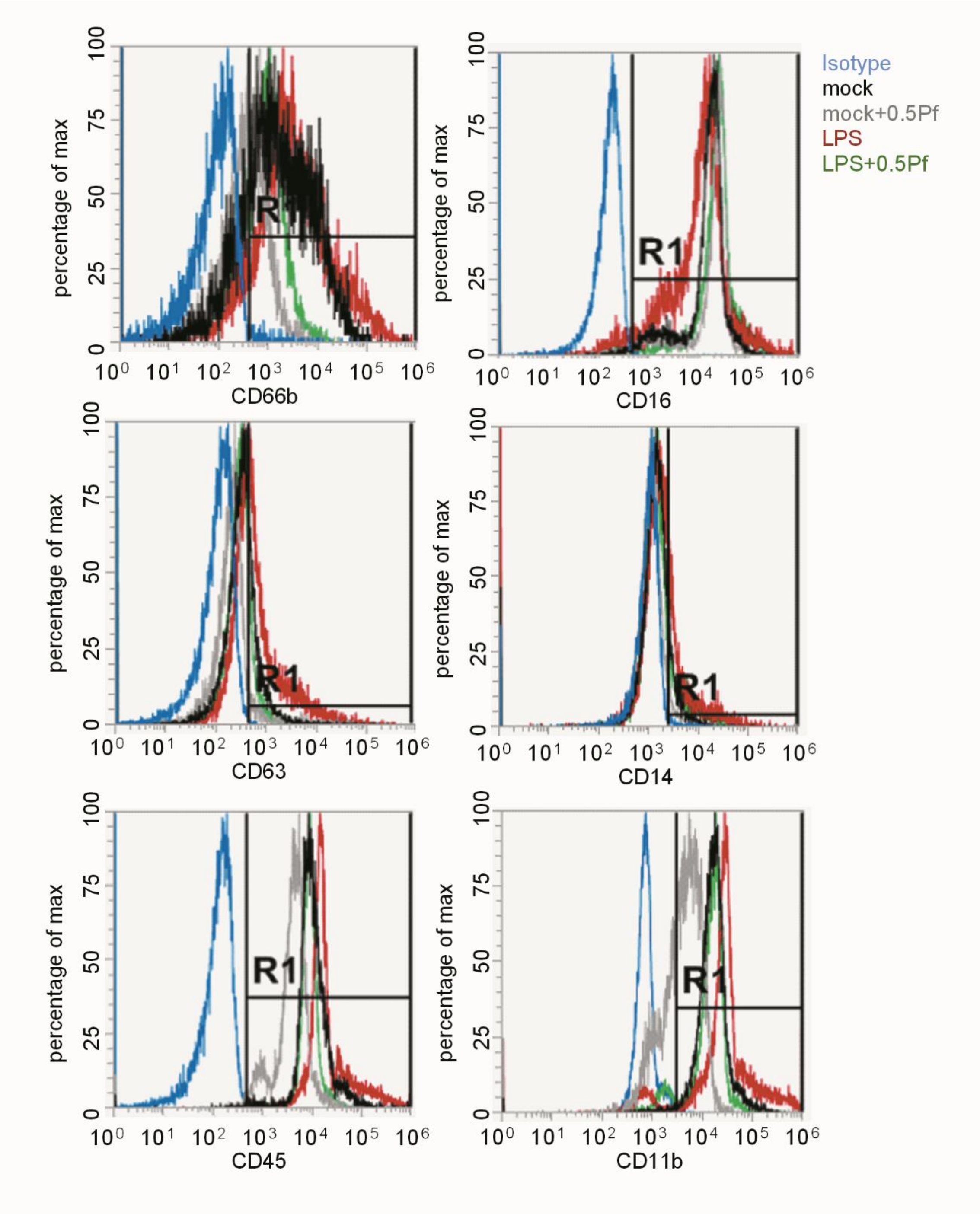

Figure S5. Representative histograms for neutrophil degranulation markers measure in the Figure 5 of the main-manuscript.

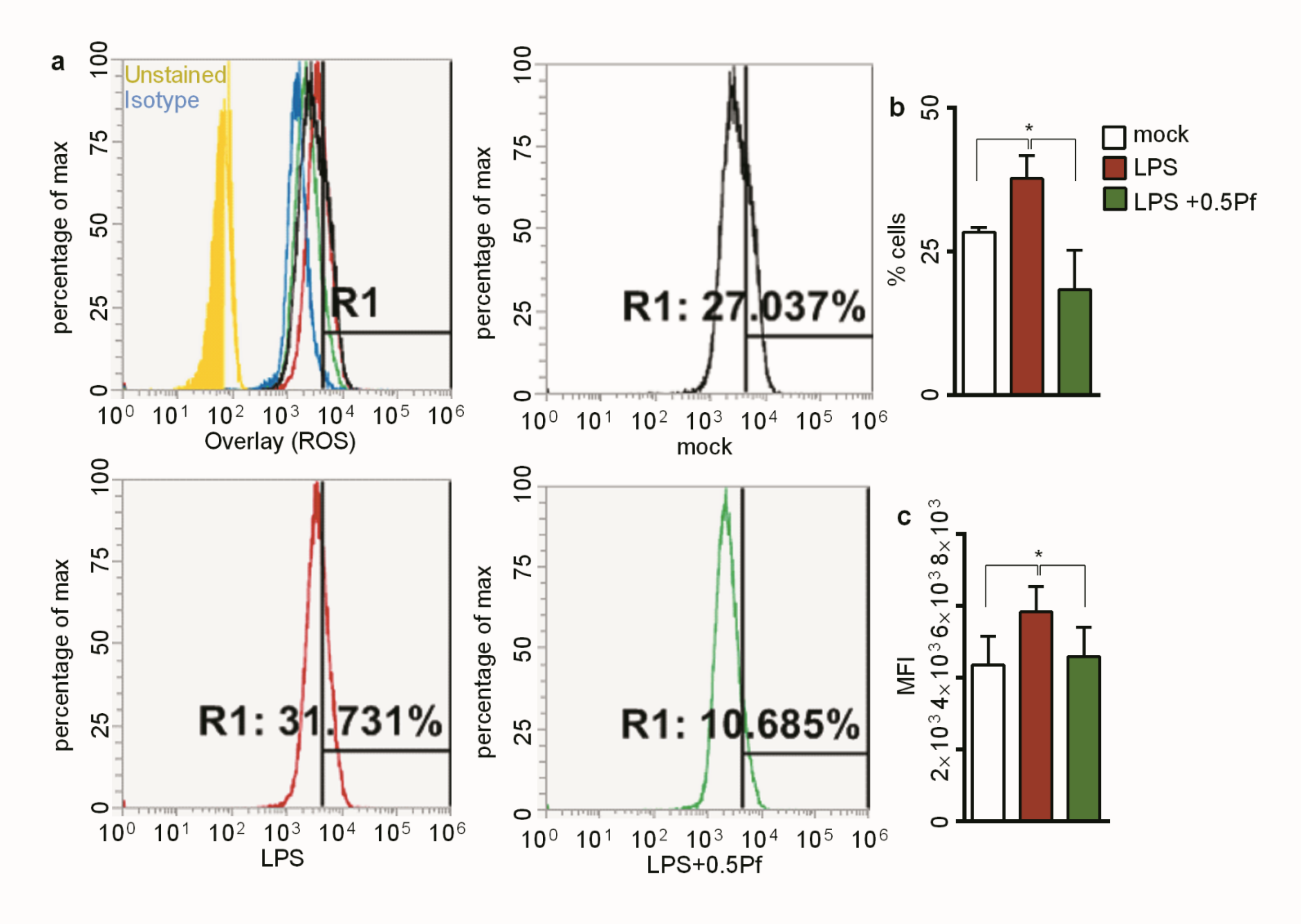

Figure S6. Phospho-p38MAPK expression by neutrophils. Mock or LPS-activated neutrophils were treated with ± 0.5 mg/ml Pf and incubated for 3 hours. Cells were collected and stained by the rabbit anti-human Phopsho-p38 primary antibody (Cell Signaling technology; USA), followed by the AF-488-anti-rabbit secondary antibody (Life Technologies; USA) and analyzed by flow cytometry. (a) Representative histograms for phosphorylated p38MAPK in neutrophils of various conditions. % positive cells (b) and MFI (c) of phosphorylated p38MAPK are graphed with mean± SEM obtained from 3 different experiments done in duplicates. * represents significant difference (*p*-value ≤ 0.05; two-way ANOVA) between various treatment groups.

Reference:

1 Calzetti, F., Tamassia, N., Arruda-Silva, F., Gasperini, S. & Cassatella, M. A. The importance of being "pure" neutrophils. *J Allergy Clin Immunol* **139**, 352-355 e356, doi:10.1016/j.jaci.2016.06.025 (2017).
